# Characterization and phylogenetic analysis of the complete mitochondrial genome of the pathogenic fungus *Ilyonectria destructans*

**DOI:** 10.1038/s41598-022-05428-z

**Published:** 2022-02-11

**Authors:** Piotr Androsiuk, Adam Okorski, Łukasz Paukszto, Jan Paweł Jastrzębski, Sławomir Ciesielski, Agnieszka Pszczółkowska

**Affiliations:** 1 Department of Plant Physiology, Genetics and Biotechnology, Faculty of Biology and Biotechnology, University of Warmia and Mazury in Olsztyn, ul. M. Oczapowskiego 1A, 10-719 Olsztyn, Poland; 2 Department of Entomology, Phytopathology and Molecular Diagnostics, Faculty of Agriculture and Forestry, University of Warmia and Mazury in Olsztyn, ul. Prawocheńskiego 17, 10-720 Olsztyn, Poland; 3 Department of Environmental Biotechnology, Faculty of Geoengineering, University of Warmia and Mazury in Olsztyn, Słoneczna 45G, 10-719 Olsztyn, Poland

**Keywords:** Fungal genetics, Mitochondrial genome, Next-generation sequencing, Genetic variation

## Abstract

*Ilyonectria destructans* is a pathogenic fungus causing root rot and other symptoms on trees and many crops. This paper analyses the mitochondrial genome of *I. destructans* and compares it with other published Nectriaceae mitogenomes. The *I. destructans* mitogenome appears as a circular DNA molecule of 42,895 bp and an overall GC content of 28.23%. It contains 28 protein-coding genes (15 core protein genes and 13 free-standing ORFs), two rRNAs and 27 tRNAs. The gene content and order were found to be conserved in the mitogenome of *I. destructans* and other Nectriaceae, although the genome size varies because of the variation in the number and length of intergenic regions and introns. For most core protein-coding genes in Nectriaceae species, Ka/Ks < 1 indicates purifying selection. Among some Nectriaceae representatives, only the *rps3* gene was found under positive selection. Phylogenetic analyses based on nucleotide sequences of 15 protein-coding genes divided 45 Hypocreales species into six major clades matching the families Bionectriaceae, Cordycipitaceae, Clavicipitaceae, Ophiocordycipitaceae, Hypocreaceae and Nectriaceae. *I. destructans* appeared as a sister species to unidentified *Ilyonectia* sp., closely related to *C. ilicicola*, *N. cinnabarina* and a clad of ten *Fusarium* species and *G. moniliformis*. The complete mitogenome of *I. destructans* reported in the current paper will facilitate the study of epidemiology, biology, genetic diversity of the species and the evolution of family Nectriace and the Hypocreales order.

## Introduction

The genus *Ilyonectria* is one of the fungi genera with *Cylindrocarpon*-like anamorphs. Apart from *Ilyonectria*, four more genera (*Campylocarpon*, *Thelonectria*, *Rugonectria* and *Noncertain *sensu stricto) were distinguished from *Neonectria* based on a multilocus phylogenetic analysis^1^. Later on, *Neonectria radicicola*, a telemorph of *Cylindrocarpon destructans,* was annotated as a type of *Ilyonectria* (*I. radicicola*) and later renamed as *Ilyonectria destructans* (Zinssm.) Rossman, L. Lombard, and Crous^2^. I. *destructans* and its *Cylindrocarpon*-like anamorph represent a species complex of cosmopolitan soil fungi which usually form chlamydospores that allow them to survive for long dormancy periods. They are also pathogens associated with cankers, root rots and black foot disease on a wide range of hosts that include both herbaceous and woody plants^1,3^. According to Lilja et al.^4^, *I. destructans* (anamorph *Cylindrocarpon destructans*), and other *Cylindrocarpon* species, like *C. cylindroides*, *C. didymum, C. magnusiarum, C. obtusisporum*, and *C. pineum,* were reported in seedlings of *Pinus sylvestris* and *Picea abies* in Finnish nurseries. Mora-Sala et al.^5^ also reported the occurrence of species belonging to the genera *Cylindrodendrum, Dactylonectria* and *Ilyonectria* associated with seedlings of diverse hosts showing decline symptoms in forest nurseries in Spain. Sánchez et al.^6^ showed that *Dactylonectria* and *Ilyonectria* species cause black foot disease in Andean blackberry. Moreover, *Cylindrocarpon* root rot may be responsible for losses up to 30% on ginseng (*Panax quinquefolium*)^7^ and plays an important role in black foot rot of grapevines^8^. Species of this genus can also occur as saprophytes colonizing dead or dying plants and as endophytes^9–12^. The considerable variation among taxa and the wide range of *Cylindrocarpon*-like species associated with root disease contribute to difficulties in identifying them^13^. A large number of studies have been related to the morphological and molecular characteristics of *Cylindrocarpon*-like species^5,7,14–16^.

Since the 1960s, when it was discovered that mitochondria contain DNA and their own RNA translation system^17^, huge progress in the molecular genetics of these organelles has been observed. Recently, the emergence of high throughput sequencing technology has enabled a rapid increase in the number of available genomic sequences. Among them, mitochondrial genomes became an ideal region for further studies of taxonomy, phylogenetics and evolutionary biology of Eukaryota, including fungi^18–20^. Mitochondrial genomes also became a source of sequences commonly used for the development of specific primers allowing the discrimination of particular fungi species or strains^21,22^. The organization, structure and content of mitochondrial genomes have been intensively studied, especially in animals^23^. This preference is also observed in the number of mt genomes deposited in the Organelle Genome Resources database at NCBI server (NCBI): of the total number of 12,996 mitochondrial genomes, 11,311 represent animals, while the second position in this ranking is occupied by fungi with only 894 mt genomes, 688 of which belong to Ascomycetes (data valid for October 2021). Generally, fungal mt genomes are single circular double-stranded DNA molecules, which commonly encode 14 genes associated with electron transport and oxidative phosphorylation (*atp6, 8, 9*; *cob*, *cox1-3*, *nad1-6*, and *nad4L*), *rps3* gene for transcriptional regulation^24^, small and large subunit of mitochondrial rRNAs (*rns* and *rnl*, respectively), and a group of 22–36 tRNAs^25,26^. Comparative analyses showed that although fungal mt genomes are characterized by rather conserved gene content, they vary considerably in their order as well as the complete size of the genome^27^.

In this study, the first complete sequence of the mitochondrial genome of I. *destructans* was assembled and annotated. A comparative analysis of the gene content, gene order and genome organization of *I. destructans* mitogenome and other fungi representing the Nectriaceae (Hypocreales) family was also performed. Additionally, the current study provided insights into the evolution and dynamics of protein-coding sequences within the Nectriaceae family and revealed the phylogenetic status of *I. destructans* (among other Hypocreales members) based on a combined set of mitochondrial genes.

## Results

### Genome size and organization

**Figure 1:**
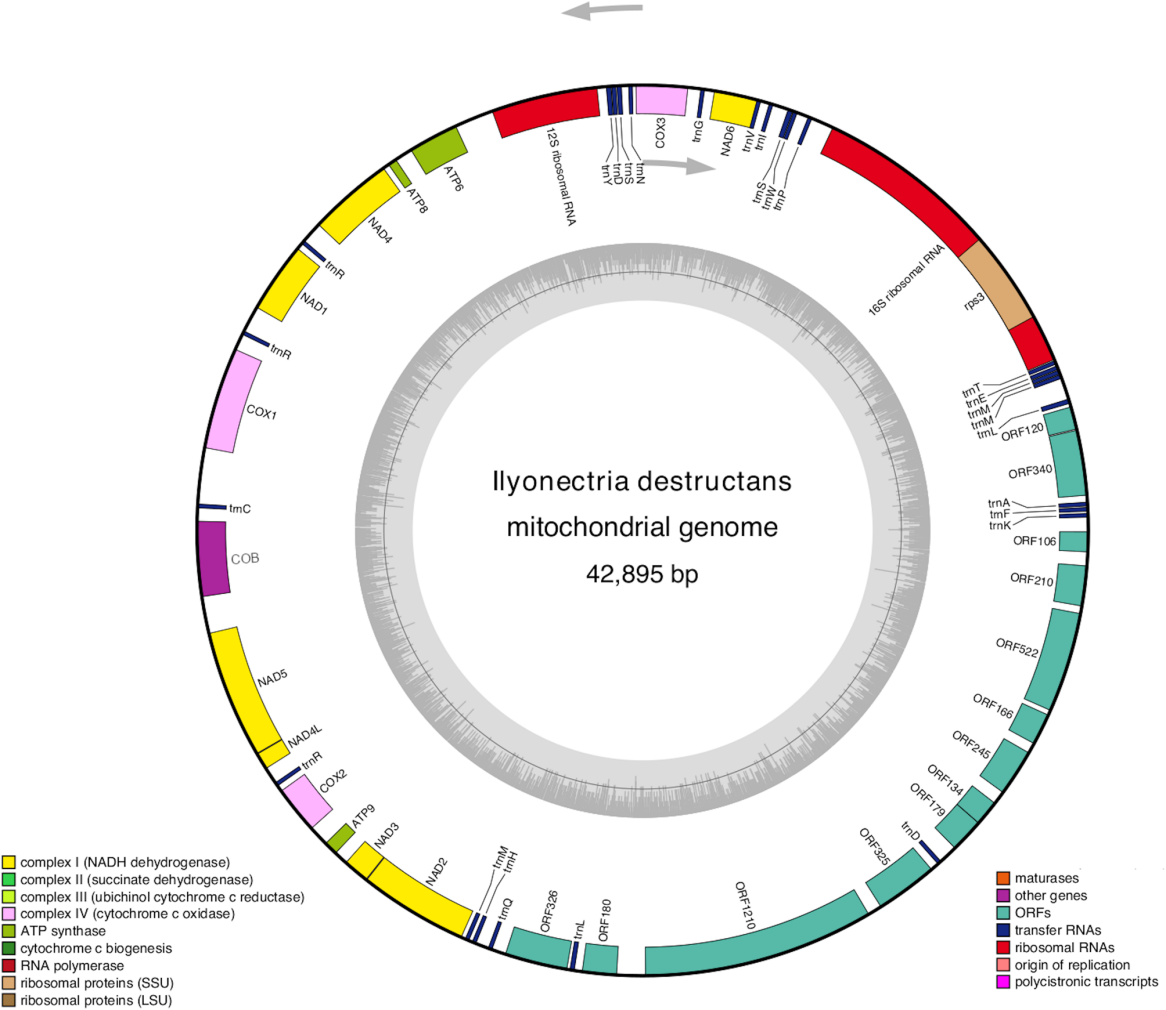
Circular map of the mitochondrial genome of *Ilyonectria destructans*. Different functional gene groups are color-coded. Genes drawn inside the circle are transcribed clockwise (indicated by arrow). GC content variations is shown in the middle circle. Gene map was generated with the OrganellarGenomeDRAW (OGDRAW) 1.3.1. (https://chlorobox.mpimp-golm.mpg.de/OGDraw.html).

Sequencing of the *Ilyonectria destructans* mitochondrial genome yielded 7,901,604 raw reads, out of which 70,052 were mapped to the reference genome of *Nectria cinnabarina* (GenBank ID: KT731105) with 312 × average coverage (max coverage 1,343). The complete mitochondrial genome of I. *destructans* represents a circular molecule with a size of 42,895 bp and an overall GC content of 28.23% (Fig. 1). A total of 28 protein-coding genes were identified in the mitogenome of *I. destructans* (Table 1). These include 14 protein-coding genes involved in oxidative phosphorylation: seven subunits of the electron transport complex I (*nad1*, *nad2*, *nad3*, *nad4*, *nad4L*, *nad5*, and *nad6*), one subunit of complex III (*cob*), three subunits of complex IV (*cox1*, *cox2*, and *cox3*) and three subunits of the ATP-synthase complex (*atp6*, *atp8*, and *atp9*). Moreover, the *rps3* gene, which encodes the 40S ribosomal protein S3, as well as genes for large and small ribosomal RNA (*rnl* and *rns*, respectively), were annotated. The sequence for the *rps3* gene was located within the intron of *rnl.* Furthermore, apart from the mentioned above genes commonly found in mt genomes of most fungi, 13 free-standing, intergenic ORFs were identified, out of which two were predicted to encode proteins that exhibited similarity to homing endonucleases from the LAGLIDADG (ORF340) and GIY-YIG (ORF326) families. Additionally, ORF1210 appeared to be similar to DNA polymerase type B protein. In the case of identified DNA polymerase gene, at the amino acid level it showed significant homology to the conserved protein family Pfam03175. In the aligned part, sequence identity was 35.7%. In the case of the remaining ORFs, no putative function was assigned to any of them, as no conservative motifs were identified within them.

**Table 1:** List of annotated mitochondrial genes in *Ilyonectria destructans.*

Gene	Position	Length (bp)	Start	Stop	Gene	Position	Length (bp)	Start	Stop
*trnK*-TTT	195–267	73			*nad1*	16,764–17,873	1110	ATG	TAA
*trnF*-GAA	287–359	73			*trnR*-UCU	18,273–18,343	71		
*trnA*-AGC	368–439	72			*cox1*	18,586–20,178	1593	ATG	TAA
ORF340	533–1555	1023	TTA	TAG	*trnC*-GCA	21,038–21,110	73		
ORF120	1562–1924	363	ATG	TAG	*cob*	21,299–22,471	1173	ATG	TAA
*trnL*-UAA	1969–2050	82			*nad5*	23,033–25,033	2001	ATG	TAA
*trnM*-CAU	2372–2444	73			*nad4L*	25,033–25,302	270	ATG	TAA
*trnM*-CAU	2445–2516	72			*trnR*-ACG	25,552–25,622	71		
*trnE*-UUC	2518–2589	72			*cox2*	25,708–26,457	750	ATG	TAA
*trnT*-UGU	2,605–2675	71			*atp9*	26,762–26,986	225	ATG	TAA
*rnl*	2681–3274, 5226–7720	3089			*nad3*	27,191–27,604	414	ATG	TAG
*rps3*	3412–4875	1464	ATA	TAA	*nad2*	27,605–29,314	1710	ATG	TAA
*trnP*-UGG	8058–8130	73			*trnM*-CAU	29,369–29,441	73		
*trnW*-UCA	8300–8371	72			*trnH*-GUG	29,487–29,559	73		
*trnS*-UGA	8372–8459	88			*trnQ*-UUG	29,750–29,822	73		
*trnI*-GAU	8691–8762	72			ORF326	30,029–31,009	981	ATT	TAG
*trnV*-UAC	8886–8957	72			*trnL*-UAG	31,045–31,125	81		
*nad6*	8914–9624	711	ATG	TAA	ORF180	31,236–31,778	543	ATG	TAA
*trnG*-UCC	9765–9836	72			ORF1210	32,209–35,808	3600	TTA	TAA
*cox3*	10,024–10,833	810	ATG	TAA	ORF325	36,016–36,993	978	ATG	TAG
*trnN*-GUU	10,876–10,946	71			*trnD*-AUC	37,110–37,183	74		
*trnS*-GCU	11,044–11,124	81			ORF179	37,467–38,006	540	ATG	TAA
*trnD-*GTC	11,131–11,204	74			ORF134	37,996–38,400	405	ATG	TAA
*trnY*-GUA	11,205–11,288	84			ORF245	38,595–39,332	738	ATG	TAA
*rns*	11,419–13,078	1660			ORF166	39,483–39,983	501	ATG	TAA
*atp6*	13,673–14,461	789	ATG	TAA	ORF522	40,051–41,619	1569	ATG	TAG
*atp8*	14,725–14,871	147	ATG	TAA	ORF210	41,726–42,358	633	ATG	TAA
*nad4*	14,956–16,287	1332	TTA	TAA	ORF106	42,562–42,883	321	ATG	TAG
*trnR*-ACG	16,605–16,675	71							

The *I. destructans* mitogenome also included 27 tRNA genes (*trn*) that recognize codons for all amino acids. There were 25 tRNA genes with a single copy and an additional two which had two (*trnR*-ACG) or three copies (*trnM*-CAU). All tRNAs fold into the typical clover-leaf structure. However, for five tRNA genes (*trnL-*UAA, *trnL-*UAG*, trnS-*UGA*, trnS-*GCU and *trnY-*GUA), an additional, variable arm was observed, located between the stem with an anticodon loop and a stem with a T loop (Supplementary Figure S1). The majority of tRNA genes were located within three tRNA clusters. The main tRNA cluster (cluster 1) was located between *rnl* and *nad2* where 13 tRNA gene sequences can be found (TEMMLAFKDLQHM), separated by ORFs sequences. Cluster 2 (VISWP) was located downstream *nad6*, whereas cluster 3 (YDSN) was downstream *rns*. The remaining tRNA genes were scattered throughout the mitogenome sequence as solitary genes placed between other coding sequences: *trnR*-UCU between *cox1* and *nad1*, *trnG*-UCC between *cox3* and *nad6*, *trnC*-GCA between *cob* and *cox1*, whereas two copies of *trnR*-ACG could be found in two locations—between *nad1* and *nad4*, and between *cox2* and *nad4L*.

**Figure 2:**
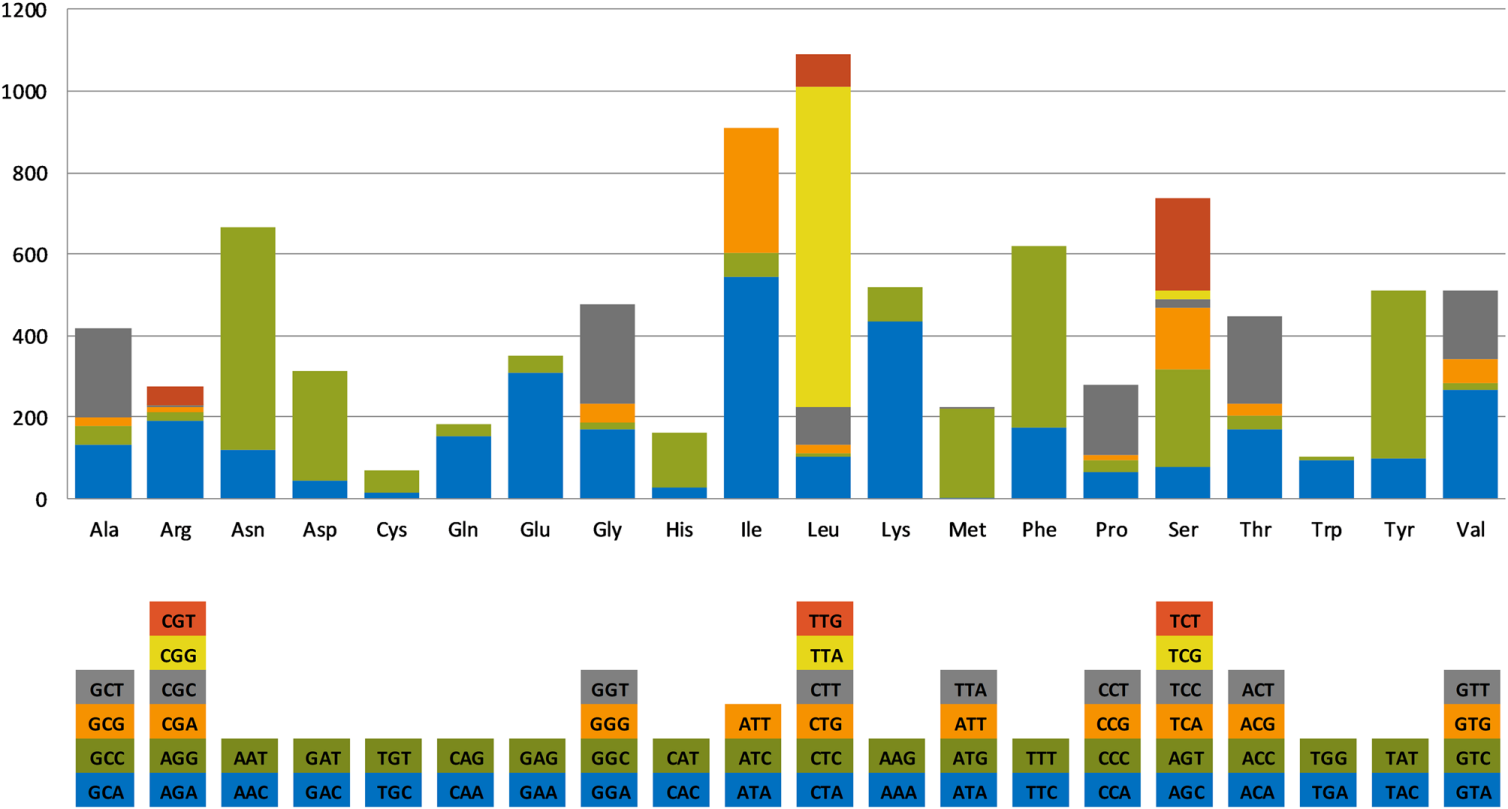
Codon usage in the mitochondrial genome of *Ilyonectria destructans*. Count of codon usage is plotted on the y-axis.

Only one gene (*rnl*) within *I. destructans* mitogenome had an intron, which contained the sequence of *rps3*. Furthermore, the mitogenome of *I. destructans* contained two pairs of overlapping genes: *trnV*-UAC/*nad6* overlapped by 43 nucleotides and ORF179/ORF134 overlapped by 10 nt. All of the 55 annotated mitochondrial genes were located on the same (sense) strand. *I. destructans* mitochondrial DNA was translated using Mitochondrial Genetic Code 4 (Mold, Protozoan). The majority of conserved protein-coding genes started with the typical ATG codon and terminated with TAA. However, in the case of the *nad4* sequence, an alternative start codon TTA was observed, whereas TAG was found as an alternative stop codon for *nad3*. Among the 13 ORFs, three started with non-canonical initiation codon TAA in the case of ORF340 and ORF1210, and ATT in the case of ORF326. Finally, eight ORFs ended with TAA, and the terminal codons of the remaining five were TAG (ORF340, ORF120, ORF 326, ORF325 and ORF 106). The codon usage analysis, which included both conserved protein-coding genes and ORFs, indicated that the most frequently used codons in *I. destructans* mitogenome were TAA (8.83% for lysine), AAT (6.14% for N), ATA (6.10% for I), TTT (5.00% for F), AAA (4.91% for K) and TAT (4.66% for Y) (Fig. 2, Supplementary Table S1). These six codons accounted for almost 36% of all codons, which contributed to the high AT content (71.77%) of the *I. destructans* mitochondrial genome.

### Repeated elements

REPuter software was used to identify 28 repetitive sequences with lengths ranging from 30 to 98 bp and sequence identities greater than 90% (Supplementary Table S2a) within the *I. destructans* mitochondrial genome. Most of the identified repeated sequences represented forward repeats (18), whereas palindromic (7) and reverse (2) repeats were in the minority, and complement repeats were not detected. Most of the repeat sizes were between 30 and 35 bp (85.7%), followed by 45–57 bp (10.7%) with one 98 bp repeat (3.6%). The repetitive sequences were unevenly distributed, mostly (78.6%) within intergenic regions of the mitogenome, while the remaining 12.4% were located in the sequences coding tRNA. The pattern of repeat sequences within the *I. destructans* mitochondrial genome was compared with the rate of repeats estimated for unidentified *Ilyonectria* species whose complete sequence of mitogenome is accessible at an NCBI server under the accession number MH924828. In that case, 13 repetitive sequences with lengths ranging from 30 to 34 bp and sequence identities greater than 90% were found. Only two types of repeated sequences were identified: 11 forward repeats and two palindromic repeats, which were located almost exclusively within the intergenic regions of the mitogenome (Supplementary Table S2b).

The distribution and types of microsatellites were also studied in the *I. destructans* mitochondrial genome. Generally, the *I. destructans* mitogenome appeared as rather poor in terms of SSR content, as only 14 of such elements were identified. Most of the SSRs represented trinucleotide repeats (5) with two ATA/TAT and ATT/TAA motifs and one AAT/TTA. Among dinucleotide SSRs, only three repeats were detected, AT/TA appeared two times and AC/TG only once. Moreover, three tetranucleotide repeats (TGCA/ACGT, TTAA/AATT, AGCA/TCGT) and three hexanucleotide repeats (AAGCTA/TTCGAT, TTATTC/AATAAG, TTTTCT/AAAAGA) were found. No mononucleotide repeats or pentanucleotide repeats were identified in the *I. destructans* mitogenome. The SSRs can be distributed across three different genomic regions: exons, introns, and intergenic spacers (IGS). In the current study, 71.4% of SSRs were found within the IGS (Supplementary Table S3a). The distribution of SSRs within the mitogenome of unidentified *Ilyonectria* sp. (MH924828) was also analyzed. Twelve SSRs were found, which include two dinucleotide repeats, two trinucleotide repeats, five tetranucleotide repeats and three hexanucleotide repeats, and mono- and pentanucleotide repeats were not detected. Among dinucleotide repeats, only one motif was observed (AC/TG), which appeared two times. For trinucleotide SSRs, two motifs were observed (AAT/TTA and ATC/TAG). Three repeat motifs (AAAT/TTTA, ATGC/TACG, AAGC/TTCG) for tetranucleotide SSRs and three motifs (AAACGT/TTTGCA, AGATAT/TCTATA, AAATAT/TTTATA) for hexanucleotide SSRs were found. Similar to *I. destructans*, SSRs were also found here predominantly within IGS (66.7%) (Supplementary Table S3b).

### Comparative analysis of *I. destructans* mitogenome to other Nectriaceae genomes

The size of the *I. destructans* mitogenome is the fourth-smallest genome among the 15 mitochondrial genomes yet sequenced for Nectriaceae (Table 2). The GC content of the *I. destructans* mitogenome is low (28.23%) and appeared as the lowest among the studied Nectriaceae genomes. Comparative analyses of mitochondrial genomes of 15 Nectriaceae species revealed that all of them contain an almost identical set of coding sequences, which included 14 proteins involved in oxidative phosphorylation (*nad1*, *nad2*, *nad3*, *nad4*, *nad4L*, *nad5*, *nad6, cob, cox1*, *cox2*, *cox3*, *atp6*, *atp8*, *atp9*), the *rps3* gene, which encodes the 40S ribosomal protein S3, genes for large and small ribosomal RNA (*rnl* and *rns*, respectively) and 25–28 tRNAs. The order of the above-mentioned protein-coding genes and two ribosomal RNA genes is highly conservative among all Nectriaceae representatives, with the *rps3* sequence located within the intron of *rnl*. The highest number of introns was described for *F. pseudograminearum* (42), *F. culmorum* (37), and *F. graminearum* (34), followed by *F. gerlachii* (33) and *F. cerealis* (33). The above-mentioned *Fusarium* species were the top five Nectriaceae representatives when the mitogenome size is considered (given in descending order). The highest number of free-standing ORFs was found in the *I. destructans* (13) mitogenome, followed by *F. cerealis* (6) and *F. culmorum* (6). For four Nectriaceae representatives, no free-standing ORFs were annotated (*F. bambusae, F. circinatum, F. oxysporium* and *N. cinnabarina*).

**Table 2:** Basic characteristic of Nectriaceae mitogenomes. Species names arranged alphabetically.

Species	Accession number	Size (bp)	GC%	Conserved PCGs	tRNAs	Introns	Free-standing ORFs	intronic ORFs
*Calonectria ilicicola*	NC_046826	39,891	28.48	15	26	5	2	4
*Fusarium bambusae*	NC_044490	63,593	31.92	15	27	11	0	0
*Fusarium cerealis*	NC_046567	93,160	31.74	15	28	33	6	31
*Fusarium circinatum*	NC_022681	67,109	31.45	15	27	14	0	15
*Fusarium commune*	NC_036106	47,526	32.42	15	26	3	2	1
*Fusarium culmorum*	NC_026993	103,844	31.68	15	28	37	6	39
*Fusarium gerlachii*	NC_025928	93,428	31.91	15	28	33	5	33
*Fusarium greminearum*	NC_009493	95,676	31.84	15	28	34	1	33
*Fusarium pseudograminearum*	NC_046566	110,525	31.64	15	28	42	4	46
*Fusarium solani*	NC_016680	62,978	28.88	15	25	15	2	13
*Fusarium oxysporium*	NC_017930	34,477	30.98	15	25	2	0	1
*Giberella moniliformis*	NC_016687	53,753	32.61	15	27	5	2	4
*Ilyonectria destructans*	NC_030340	42,895	28.23	15	27	1	13	0
*Ilyonectria* sp.	MH924828	34,584	28.76	15	25	2	2	1
*Nectria cinnabarina*	NC_030252	69,895	28.71	15	25	17	0	0

PCG's-Protein Coding Genes; ORFs—Open Reading Frames.

In order to check the level of nucleotide sequence variation between the sampled mitogenomes of Nectriaceae representatives, the mVISTA program was used to align the sequences with the annotation of *I. destructans* as a reference. The result of the alignment indicated that analyzed mitogenomes are generally conserved, although some level of variation was detected: protein-coding sequences appeared as more conserved than the non-coding regions (Supplementary Figure S2). The application of MAUVE software did not reveal any sign of structural rearrangements within the analyzed mitogenomes (Supplementary Figure S3).

**Figure 3:**
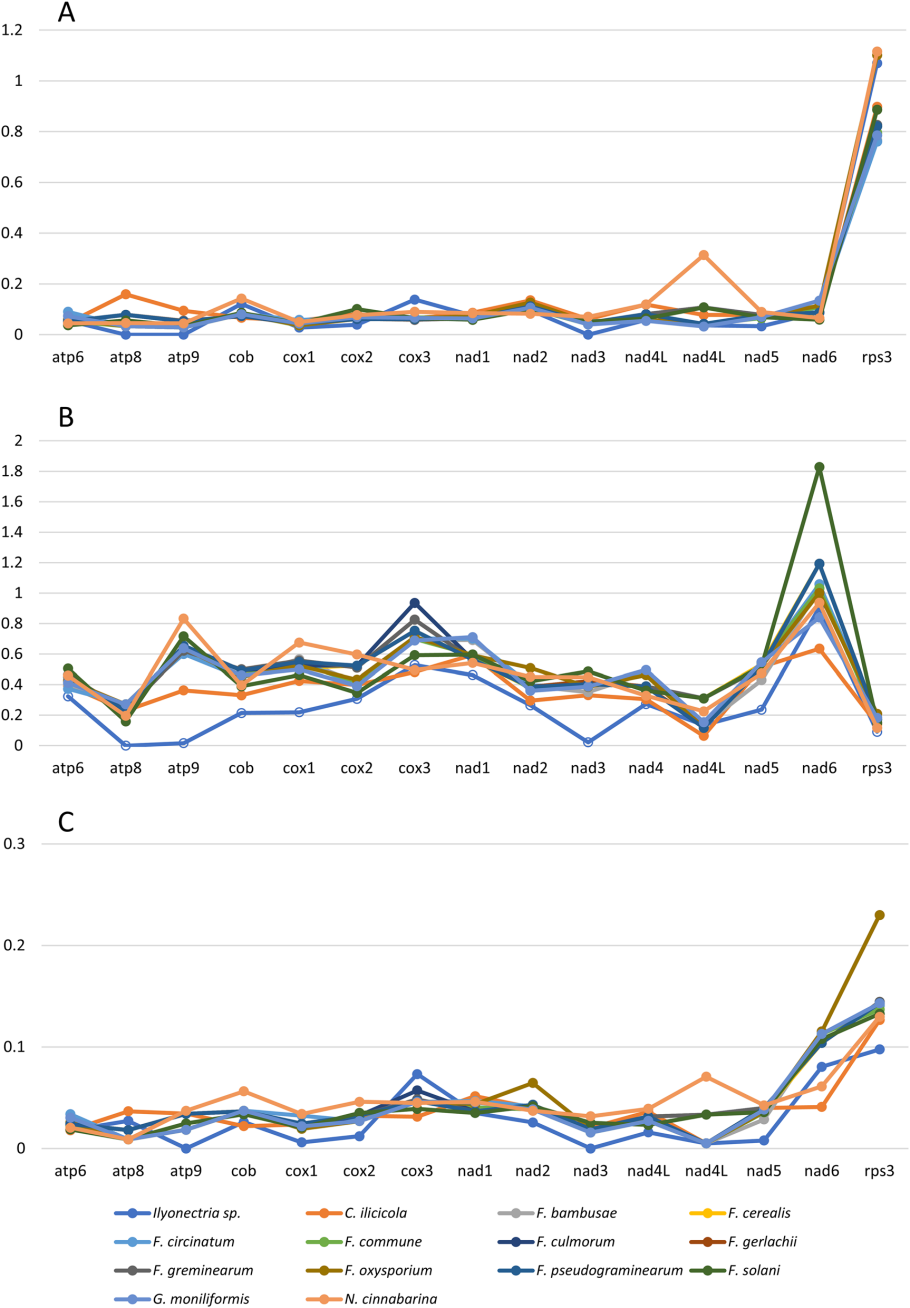
The evolution and dynamic of mitochondrial protein-coding sequences between 15 species representing family Nectriaceae. The mt genome of *Ilyonectria destructans* was set as a reference. (**A**) Gene specific Ka/Ks ratios; (**B**) Synonymous (Ks) substitution rates; (**C**) Non-synonymous (Ka) substitution rates.

In order to detect the protein-coding genes that were under selective pressure, the synonymous (Ks) and non-synonymous (Ka) substitution rate, as well as the Ka/Ks ratio, were calculated using DnaSP among the mitogenomes of 15 Nectriaceae representatives. Genes with non-applicable (NA) Ka/Ks ratios were changed to zero. The results revealed that the Ka/Ks ratio was < 1 in most of the genes with the exception of *rps3* for *I. destructans* vs. *Ilyonectria* sp., *I. destructans* vs. *F. oxysporium* and *I. destructans* vs. *N. cinnabarina* (1.0701, 1.1015 and 1.1161, respectively) (Fig. 3, Supplementary Table S4a). When the remaining genes were considered, the Ka/Ks ratio in all analyzed species did not exceed the value of 0.3138, which was noted for *nad4L* in *N. cinnabarina*. Further analysis revealed that the substitution rate varied substantially, with Ka and Ks values ranging from zero to 0.23 and from zero to 1.8276, respectively. The highest average synonymous substitution rate (average Ks = 1.1017) was observed for the *nad6* gene, whereas the lowest average Ks (0.1701) was noted for *rps3*. The highest average non-synonymous (Ka) substitution rate was observed for *rps3* (average Ka = 0.1461), whereas the lowest was noted for *atp8* (0.0146). Summing up, Ka/Ks ratio values showed that the majority of genes undergo purifying selection and only *rps3* was under positive selection. In order to test whether traces of positive selection could be observed for the *rps3* in all analyzed mitogenomes, the Ka/Ks ratio for the gene was estimated among all Nectriaceae species combinations (Supplementary Table S4b). In 12 out of 105 combinations, the Ka/Ks ratio was higher than 1.0, suggesting adaptation to environmental conditions. Moreover, in 39 other cases, the Ka/Ks ratio was slightly below one (> 0.8), whereas for the next 23 cases, the Ka/Ks ratio was between 0.8 and 0.7, which may indicate at least some role of positive selection affecting *rps3* in the acceleration of the substitution rate.

### Phylogenetic analysis

**Figure 4:**
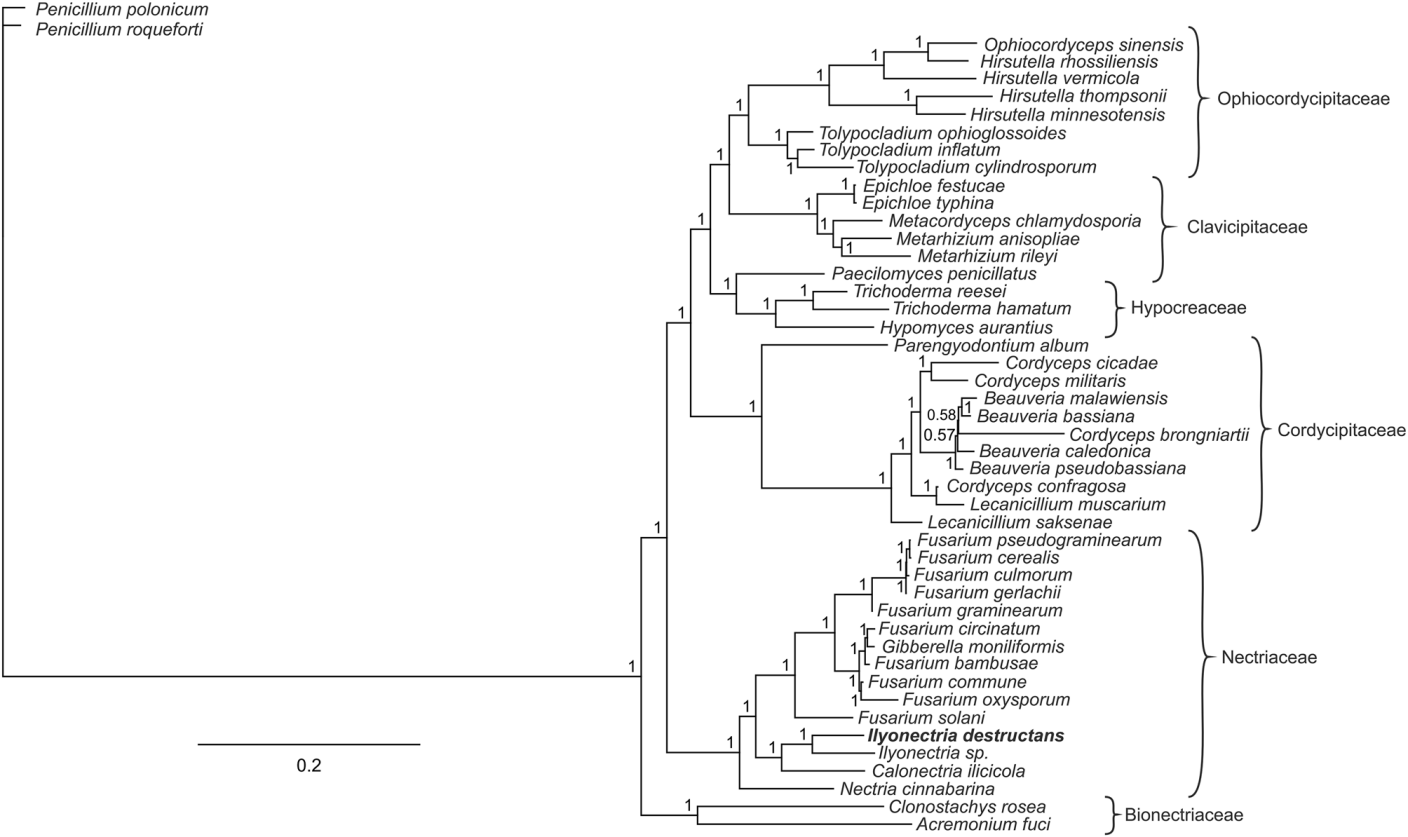
Phylogenetic tree based on sequences of sheared 15 protein-coding genes from 45 fungi species representing Hypocreales and two *Penicillium* species (outgroup) using Bayesian posterior probabilities (PP). Bayesian PP are given at each node.

The phylogenetic tree was reconstructed based on concatenated nucleotide sequences of 15 protein-coding genes shared by 45 species representing Hypocreales and two Penicillium species used as the outgroup. Both BI and ML methods generated phylogenetic trees with consistent topology. All of the recovered clades of the BI tree were characterized by very high nodal support values (only two nodes had a Bayesian posterior probability value below 1.0). Based on the phylogenetic analysis, the 45 Hypocreales species could be divided into six major clades matching the Bionectriaceae, Cordycipitaceae, Clavicipitaceae, Ophiocordycipitaceae, Hypocreaceae and Nectriaceae families (Fig. 4). The 15 Nectriaceae species were divided into three clades. *N. cinnabarina* was the first single clad. The second clad consisted of a pair of *Ilyonectria species* (*I. destructans* and unidentified *Ilyonectria* sp.) and a separate branch for *Calonectria ilicicola*. The genus *Fusarium*, containing ten *Fusarium* species and *G. moniliformis* (anamorph *Fusarium verticillioides* (Sacc.) Nirenberg), formed the third clad. The most distinct position on the dendrogram was occupied by *Penicillium roqueforti* and *P. polonicum*, used here as an outgroup.

## Discussion

The present study is the first report of the complete mitochondrial genome sequence of the species representing the genus *Ilyonectria* from the Nectriaceae family. The application of next-generation sequencing technology allowed the size of the *Ilyonectria destructans* mitogenome to be precisely estimated (42,895 bp). As far as it is known, there is only one available record of a complete mitogenome of *Ilyonectria* in the GenBank (MH924828;^28^). Unfortunately, the authors^29^ were not able to precisely identify the species and reported it as *Ilyonectria* sp. This circular mitogenome had a total length of 34,584 bp and contained 18 protein-coding genes, two ribosomal RNA genes (*rns* and *rnl*) and 25 tRNA genes. Nevertheless, the current study revealed that mitogenomes of *Ilyonectria destructans* and *Ilyonectria* sp. represent two different *Ilyonectria* species, based on their size, structure and content.

The GenBank resources currently encompass complete sequences of mitochondrial genomes of 15 representatives of Nectriaceae family, including ten *Fusarium* species, one representative of the genus *Nectria*, *Calonectria* and *Gibberella*, and two *Ilyonectria* fungi – *I. destructans* (reported in this paper) and one unidentified *Ilyonectria* species (mentioned above). The size of mitochondrial genomes for this group of fungi range from 110,525 bp for *Fusarium pseudograminearum* to 34,477 bp for *F. oxysporium*, thus *I. destructans* is one of the species with the smallest mitogenomes. It is observed that the fungal mitochondrial genomes may vary substantially in terms of their size and composition^30^. Currently, the largest fungal mitochondrial genome ever sequenced and annotated is *Golovinomyces cichoracearum* representing Ascomycota^28^. This mitogenome has a length of 332,165 bp and includes 58 genes: 30 protein-coding genes, two rRNA genes and 26 tRNA genes. The smallest fungal mitogenome was found in *Rozella allomycis*, representing Cryptomycota, and it included 14 genes: eight protein-coding genes, two rRNAs and four tRNAs^28^. Although the mitochondrial genomes of Nectriaceae showed significant variation in their sizes, the current study found high synteny among them: the gene composition and their order was quite identical and encompassed 14 protein-coding genes whose products are involved in oxidative phosphorylation, a gene for the 40S ribosomal protein S3 (*rps3*), two rRNAs and 25—28 tRNAs. GC content is among the basic characteristics of the genomes routinely reported along the sequence itself. The GC content in mt genomes of Nectriaceae varies significantly and is the highest for *Giberella moniliformis* (32.61%) and *Fusarium commune* (32.42%), whereas the smallest value of this trait is found in *I. destructans* (28.23%). For unidentified *Ilyonectria* species available at the NCBI, the GC content was quite similar (28.76%). Although it has been reported that a high GC content has a positive impact on the stability of the DNA structure^31^ and that it may be positively correlated with the genome size^32^, the current study did not reveal any correlation between GC content and size in the studied Nectriaceae mitogenomes.

According to the available literature, the number and size of introns and intergenic regions, as well as the abundance of repetitive elements or intensity of gene transfer events, have an influence on the size of fungal mitochondrial genomes^33,34^. For the studied Nectriaceae, the number and size of introns, as well as the abundance of intergenic regions, were found to be closely related to the size variations of mitogenomes: *F. pseudograminearum*, *F. culmorum*, and *F. graminearum,* which are characterized by the largest mitogenomes, also have the highest number of introns (42, 37 and 34, respectively). Furthermore, the introns were unevenly distributed among genes, with the highest frequency in *cox1*, for which up to 16 introns were found (*F. pseudograminearum*). A high intron content within the *cox1* sequence was also previously observed in other fungal mitogenomes representing both Ascomycota^35^ and Basidiomycota^36^. For *I. destructans*, only one intron within the *rnl* gene was identified, which was long enough to contain a sequence of the *rps3* gene. In mitogenome of unidentified *Ilyonectria* sp., except intron within *rnl* gene (which include sequence for *rps3*), one intron within *cob* gene was also annotated. The observed variation in the number and distribution of introns suggests that the intron loss/gain phenomena may have occurred in the evolution of Nectriaceae. Additionally, for the most analyzed Nectriaceae mitogenomes, many open reading frames were also identified, predominantly placed within the group I introns. The highest number of these elements were identified in mitogenomes of *F. pseudograminearum* (46) and *F. culmorum* (39), whereas no such element was annotated in *F. bambusae*, *N. cinnabarina* or *I. destructans*. These elements are dominated by ORFs that exhibit similarities to the homing endonucleases from the LAGLIDADG and GIY-YIG families. A much smaller number of ORFs were found within intergenic spacers, and they ranged from none (zero) in *F. bambusae, F. circinatum, F. oxysporium* and *N. cinnabarina* up to 13 of such elements in *I. destructans*. For three free-standing ORFs identified within *I. destructans* mitogenome, a putative function could be assigned: ORF 340 encodes a putative LAGLIDADG endonuclease protein, ORF 326 encodes a putative GIY-YIG endonuclease protein, whereas ORF1210 encodes a putative DNA polymerase type B protein. All three demonstrated similarity to relevant sequences in the mitochondrial genomes of other fungi and possessed conserved domain motifs. In fact, B-type DNA polymerase (Pfam 01,375) genes have been widely reported in fungal plasmids and mt DNA^37^ as well as both endonuclease proteins^38^. However, the true character of the above-mentioned proteins, i.e. whether they are active proteins or only non-functional pseudogenes, is not certain since their identification and annotation was based solely on bioinformatic analysis. For the remaining ORFs, no putative function could be assigned, as no conserved motifs were found within their sequences. Although ORFs are a common structural element of fungal mitochondrial genomes, their function quite often remains unclear^39^. The size of all ORFs in the *I. destructans* mitogenome accounts for 28.43% (12,195 bp) of its total mitogenome. In comparison, the mitochondrial genome of the unidentified *Ilyonectria* species contains three ORFs with a total length of 4683 bp, which is 13.54% of its total mitogenome. This observation shows that these elements are important factors affecting the size of mitochondrial genomes of the genus *Ilyonectria*. Moreover, the presence of such elements, their number, unique distribution patterns and characteristics all appear as a potential target for further studies on the evolution of mitogenomes or PCR-based species identification^40,41^.

Repetitive elements are known to be highly diversified in terms of their type and distribution. Since their accumulation in fungal mitogenomes may promote recombination, they are considered the main factors resulting in the high variation observed in the structure and organization of mitochondrial genomes^27^, even within one genus^42^. However, in the present study, it was found that the mitogenome of *I. destructans* contains a rather moderate number of such elements (28), among which forward, palindromic and reverse repeats were identified, distributed predominantly (78.6%) within intergenic regions. When the mitogenome of the unidentified *Ilyonectrai* sp. was considered, the number of repetitive elements was even lower (11 forward repeats and two palindromic repeats), but they share the same distribution pattern, i.e. they could be found almost exclusively within intergenic regions. A low repetitive element content could, therefore, be one of the mechanisms responsible for the lack of large-scale rearrangements within mitochondrial genomes of the genus *Ilyonectria*. Simple sequence repeats (SSRs), also known as microsatellites, represent another group of repetitive elements of the genome. SSRs, due to their high polymorphism, codominant inheritance and multi-allelic character, are widely used molecular markers in population genetics, genetic diversity or fingerprinting analysis of many organisms, including fungi^43,44^. The mitogenomes of *I. destructans* and *Ilyonectria* sp. are characterized by their low content (14 and 12, respectively). Among these SSRs, most (5/14) consisted of trinucleotide repeats (for *I. destructans*) or tetranucleotide repeats (5/12) for unidentified *Ilyonectria* sp., while di- and hexanucleotide repeats were identified with much lower frequencies. No mono- and pentanucleotide SSRs were found in either *Ilyonectria* mitogenomes. Furthermore, most of the identified SSRs are composed of motifs rich in A and T, which is congruent with previous observations of fungi^45,46^. The SSRs can be distributed across three different genomic regions: exons, introns and intergenic spacers (IGS). In the current study, the majority of SSRs identified within the mitogenome of *I. destructans* and *Ilyonectria* sp. were found within IGS (71.4% and 66.7%, respectively). Moreover, a comparison of different classes of SSRs revealed that trinucleotide repeats were the most abundant SSR class within coding sequences. Analogous observations were also reported previously^47,48^. According to the authors, SSRs located within the coding region do not cause a frame shift within the coding sequence; they are translated into amino acid repeats and, thus, may contribute to the biological function of the protein.

An analysis of synonymous and non-synonymous substitution patterns within coding sequences for mitochondrial proteins was also one of the major elements of the current study. An analysis of 15 genes of 14 species representing the Nectriaceae family showed that different proteins showed a differentiated mutation rate, but synonymous substitutions generally dominated over non-synonymous, which is in accordance with previous observations^49^. The highest average synonymous substitution rate was observed for the *nad6* gene, whereas the lowest average Ks was noted for *rps3*. This unique character of the *nad6* gene was conditioned by high synonymous substitution rate values (Ks > 1.0) estimated for most of the studied Nectriaceae representatives. Only for *N. cinnabarina*, *Ilyonectria* sp., *G. moniliformis* and *C. ilicicola* were Ks values for *nad6* slightly below 1.0. The *rps3* gene was found to be differentiated greatly among the studied fungal species due to the highest number of non-synonymous substitutions. This was accompanied by high Ka/Ks values for all *rps3* sequences in studied Nectriaceae representatives when compared to *I. destructans* used as a reference. For three species (*N. cinnabarina*, *F. oxysporium* and *Ilyonectria sp*.), the Ka/Ks values were greater than 1.0, suggesting that positive selection acts on *rps3* in the Nectriaceae family. For the remaining species, the Ka/Ks value ranged from 0.7612–0.8979, which may be a confirmation of this tendency. Similar mechanisms of *rps3* evolution (positive selection), which may result from the differentiation of life styles and environmental adaptations, were noted in various fungal lineages^19,50^, including Hypocrealaes^51,52^. For the remaining genes, the Ka/Ks ratio for all studied species did not exceed 0.3138 (*nad4L* in *N. cinnabarina*) which suggests that they undergo purifying selection.

Mitochondrial genomes have become a very popular object of studies devoted to the evolution and systematics of Eucaryotes. The usefulness of mitogenomes is especially observed in fungal phylogenetic analyses, since for many lineages it is difficult to classify them just based on their morphology due to variable or overlapping morphological characteristics^53^. Therefore, traditional taxonomic studies based on morphological analysis need to be supported with the application of molecular markers. A large number of pathogenic fungi of the genus *Cylindrocarpon*, *Fusarium* and *Cylindrocladium,* which belong to *Nectriaceae* (Hypocreales), show a taxonomic association with *C. destructans*^10^. Booth^14^ divided the species belonging to the genus *Cylindrocarpon* into four groups depending on the presence or absence of chlamydospores and microconidia: *C. magnusianum*, *C. cylindroides*, *Nectria mammoidea* and *C. destructans*. In later studies, Samuels and Brayford^3^ reviewed the existing classification based on morphology and culture characteristics and categorized *Nectria radicicola*, including the asexual generation of *C. destructans* generation in three varieties, known as var. *radicicola* (anamorph: *C. destructans* var. *destructans*), var. *coprosmae* (anamorph: *C. destructans* var. *coprosmae*) and var. *macroconidiales* (anamorph: *C. macroconidiales*). In turn, Mantiri et al.^15^ modified the Booth^14^ classification and redefined the genus *Nectria* into three clades (i.e. clade I: group *Nectria coccinea*/*galligena*; clade II: *N. mammoidea*/*veuillotiana* group; and clade III: *N. radicicola* group) based on an analysis of mitochondrial rDNA sequences and included *C. destructans* into clade III. Cabral et al.^54^, based on phylogenetic analyses of isolates deposited in culture collections as *C. destructans*, indicated that they represent many species of *Ilyonectria*, suggesting that *C. destructans* sensu stricto (originally described as *Ramularia destructans* Zinssm. from *Panax quinquefolium* L.) is rare. Phylogenetic analyses of *Cylindrocarpon destructans* isolates from Korean ginseng (*Panax ginseng*), based on variation in the sequence of nuclear internal transcribed spacer (ITS), confirmed that they belong to *Nectria/Neonectria radicicola* complex^55^. Moreover, high variation in their virulence was observed. Based on the results of virulence tests, *C. destructans* isolates originating from several Korean regions were divided into two distinct groups: the first group gathered highly pathogenic isolates (pathogenicity group II), and the second group contained isolates characterized by weak virulence (pathogenicity group I)^55^. Additionally, higher genetic variation of the second group was revealed by a mitochondrial small subunit (mt SSU) rDNA sequence analysis^55^.

Gene sequences originating from mitochondrial genomes are valuable for phylogenetics, evolutionary and population genetic studies because of their mutation rate, which is higher than in the case of nuclear coding sequences and the high number of available molecular markers^25,56^. In the present study, phylogenetic relationships were reconstructed within Hypocreales based on sequences of 15 protein-coding genes. The reliability of these results is indicated by high support rates. Phylogenetic tree topology is concordant with the actual systematics of this fungi group, recognizing six families within the order Hypocreales (Bionectriaceae, Cordycipitaceae, Clavicipitaceae, Ophiocordycipitaceae, Hypocreaceae and Nectriaceae), similar to that based on 14 mitochondrial protein-coding genes^57^ as well as a tree based on five nuclear genes^58^. According to expectations, *I. destructans* representing the Nectriaceae family appeared as a sister species to unidentified *Ilyonectia* sp., closely related to *C. ilicicola*. In the close vicinity of these three species, a separate branch for *N. cinnabarina* and a clad which included ten *Fusarium* species and *G. moniliformis* (anamorph *Fusarium verticillioides* [Sacc.] Nirenberg) were observed. Analogous phylogenetic relationships among 13 species representing the Nectriaceae family was also observed by Yang et al.^28^ based on Bayesian interference analysis of the combined set of 15 mitochondrial protein-coding genes.

## Conclusions

In conclusion, the complete mitochondrial genome of *Ilyonectria destructans* was sequenced, annotated and reported for the first time. The results of this study revealed high similarities among mitogenomes of *Ilyonectria* species as well as all representatives of the Nectriaceae family that are linked with the gene content, order and orientation. The high variation observed in the size of the mitochondrial genome among Nectriaceae fungi is, predominantly, a result of differences in intron density and the size variation of intergenic spacers. Comparative analysis of protein-coding sequences shared by representatives of Nectriaceae family revealed that purifying selection dominates over positive selection, which acts only on one gene (*rps3*). Finally, the availability of the complete mitochondrial genome of *I. destructans* provide data essential for the development of novel genetic markers suitable for exploring the epidemiology, biology, genetic diversity of the species and the evolution of the Nectriace family and the Hypocreales order.

## Methods

### Fungal isolate and DNA extraction

The *Ilyonectria destructans* (*Cylindrocarpon destructans*) isolate 2007/P/476 used in this study originated from the collection of the Department of Entomology, Phytopathology and Molecular Diagnostics, Faculty of Agriculture and Forestry, University of Warmia and Mazury in Olsztyn, Poland^59^. It was isolated from the pea roots (*Pisum sativum* L.) cv. Ramrod grown on experimental plots of the University of Warmia and Mazury in Olsztyn, located in Tomaszkowo, Poland (53°42′58.8"N 20°26′23.0"E). Since pea is an agricultural plant in Poland, no permits are required to conduct research or experiments on this species. Moreover, all local, national or international guidelines and legislation concerning research involving plants were adhered to in this study. Fungal cultures were grown in 90 mm sterile Petri dishes with PDA (Potato Dextrose Agar) medium. Total genomic DNA was isolated from mycelium (*Ilyonectria destructans)* scraped with a sterile scalpel from the surface of 14-day PDA cultures. The cultures were grated (homogenized) with pestle and mortar in liquid nitrogen according to the protocol using a Maxwell® 16 FFS Nucleic Acid Extraction System, Customs X9431 (Promega GMBH, Madison, WI, USA).

### DNA sequencing, assembly and annotation of the mitogenomes

Genome libraries were prepared from the genomic DNA using a Nextera XT kit (Illumina Inc., San Diego, CA, USA) and were sequenced on the Illumina MiSeq Platform (Illumina Inc., San Diego, CA, USA) with a 150 bp paired-end read. The trimmed reads were mapped to the reference of the complete mitochondrial genome of *Nectria cinnabarina* (GenBank ID: KT731105) using Geneious Mapper (ver. 8.0.4)^60^ with “Medium–Low Sensitivity” parameters. Reads aligned to the reference mtDNA genome were extracted and used for de novo assembly (K-mer—23–41, low coverage cut-off—5, minimum contig length—300) separately for each *Colletotrichum* species. De novo contigs were extended by mapping raw reads to the generated contigs, reassembling the contigs with mapped reads, and manually scaffolding the extended contigs (minimum sequence overlap of 50 bp and 97% overlap identity). This process was iterated five times. Finally, the reduced sequences were assembled in the circular mitochondrial genome for each of the four species. The mitochondrial genomes were annotated using MFannot and RNAweasel (https://megasun.bch.umontreal.ca/RNAweasel)^61^ as well as PlasMapper^62^ with manual adjustments. The annotations of tRNA genes were additionally verified with the tRNAscan-SE^63^, which also enabled an analysis of their putative secondary structures. In all cases, annotation was based on Mitochondrial Genetic Code 4 (Mold, Protozoan). Open reading frames (ORFs) were functionally annotated using Blast2Go Basic^64^. A physical map of the mitochondrial genome was created with OrganellarGenome-DRAW (OGDRAW)^65^. The mitochondrial genome sequence of *I. destructans* was deposited in GenBank under accession number NC_030340.

### Characteristics and comparative analysis of the mitogenomes

The size and composition of the complete sequence of *Ilyonectria destructans* mitochondrial genome were characterized. Furthermore, the *I. destructans* mitogenome gene content and order were compared to previously published mitochondrial genomes of other representatives of the Nectriaceae family. For this purpose, the complete mitogenomes of 14 Nectriaceae fungi were downloaded from GenBank (Table 2). In order to check whether the representatives of Nectriaceae family share conserved regions with *I. destructans*, a comparison of their mitochondrial genomes was performed using the mVISTA program and the Shuffle-LAGAN mode was applied^66^. *I. destructans* was set as a reference. The comparison was performed on mitogenome sequences aligned by MAFFT v7.310^67^. An analysis of the evolutionary rates of genes shared by *I. destructans* and the above-mentioned representatives of the Nectriaceae family was also performed. A group of 15 genes were selected to estimate the ratio of non-synonymous (Ka) to synonymous (Ks) substitutions. These genes were extracted and aligned separately using MAFFT v7.310. The Ka and Ks for each of the shared genes were estimated in DnaSP^68^ with *I. destructans* as a reference.

### Identification of repetitive elements

In order to detect and assess genomic repeats, the REPuter program^69^ was used. Identification of genomic repeats included forward, reverse, palindromic and complementary sequences with a minimal length of 30 bp, a Hamming distance of 3, and 90% sequence identity. Moreover, Phobos v.3.3.12^70^ was used to identify mitochondrial simple sequence repeats (SSR) or microsatellites. During the realization of this task, only perfect SSRs with a motif size of one to six nucleotide units were searched for. Furthermore, standard thresholds for the identification of mitochondrial SSRs were applied^71^, i.e. a minimum of 12 repeat units for mononucleotide SSRs, six repeat units for dinucleotide SSRs, four repeat units for trinucleotide SSRs, and three repeat units for tetra-, penta-, and hexanucleotide SSRs.

### Phylogenetic analysis

Phylogenetic analyses were performed on sequences of 15 protein-coding genes shared by 45 fungi species belonging the Hypocreales class, including 15 representatives of *Nectriaceae* family and two species of *Penicillium* as an outgroup. The appropriate sequences were downloaded from the NCBI database (Supplementary Table S5). The selected sequences were aligned in MAFFT v7.310. The trimAl tool^72^, using the heuristic method automated1, was applied to cut gaps in the alignment. Bayesian Inference (BI) and Maximum-Likelihood (ML) methods were used for genome-wide phylogenetic analyses in MrBayes v.3.2.6^73,74^ and PhyML 3.0^75^. Before BI and ML analysis, the best fitting substitution model was searched for in Mega 7^76^, and the GTR + G + I model was selected. A BI partitioning analysis was carried out to develop a majority rule consensus tree with 1 × 10^7^ generations using the Markov Chain Monte Carlo (MCMC) method. The tree sampling frequency was 1,000 generations. The first 2,500 trees were discarded as a burn-in, with a random starting tree. The ML analysis was performed in PhyML 3.0 with 1,000 bootstrap replicates.

## Supplementary Information

Supplementary Figure S1.

Supplementary Figure S2.

Supplementary Figure S3.

Supplementary Table S1.

Supplementary Table S2.

Supplementary Table S2.

Supplementary Table S3.

Supplementary Table S3.

Supplementary Table S4.

Supplementary Table S5.

## Data Availability

The complete mitochondrial genome of *Ilyonectria destructans* has been submitted to the NCBI database (https://www.ncbi.nlm.nih.gov/) under the accession number NC_030340.
