# Supplementary material for: Characterization and phylogenetic analysis of the complete mitochondrial genome of the pathogenic fungus Ilyonectria destructans
Source: Sci Rep. 2022 Feb 11;12:2359. doi: 10.1038/s41598-022-05428-z (PMC8837645; doi:10.1038/s41598-022-05428-z)
Supplement: Supplementary file 1 — Supplementary Figure S1. [file 41598_2022_5428_MOESM1_ESM.pdf]

Characterization and phylogenetic analysis of the complete mitochondrial genome of the pathogenic fungus *Ilyonectria destructans*

Piotr Androsiuk*[1], Adam Okorski[2], Łukasz Paukszto[1], Jan Paweł Jastrzębski[1], Sławomir Ciesielski[3], Agnieszka Pszczółkowska[2]

1. Department of Plant Physiology, Genetics and Biotechnology, Faculty of Biology and Biotechnology, University of Warmia and Mazury in Olsztyn, ul. M. Oczapowskiego 1A, 10-719 Olsztyn, Poland.
2. Department of Entomology, Phytopathology and Molecular Diagnostics, Faculty of Agriculture and Forestry, University of Warmia and Mazury in Olsztyn, ul. Prawocheńskiego 17, 10-720 Olsztyn, Poland.
3. University of Warmia and Mazury in Olsztyn, Faculty of Geoengineering, Department of Environmental Biotechnology, Słoneczna 45G, 10-719 Olsztyn, Poland.

* corresponding author – piotr.androsiuk@uwm.edu.pl

Figure S1. Putative secondary structures for 27 tRNA genes of *Ilyonectria destructans*. The tRNA genes are labeled with standard abbreviations.

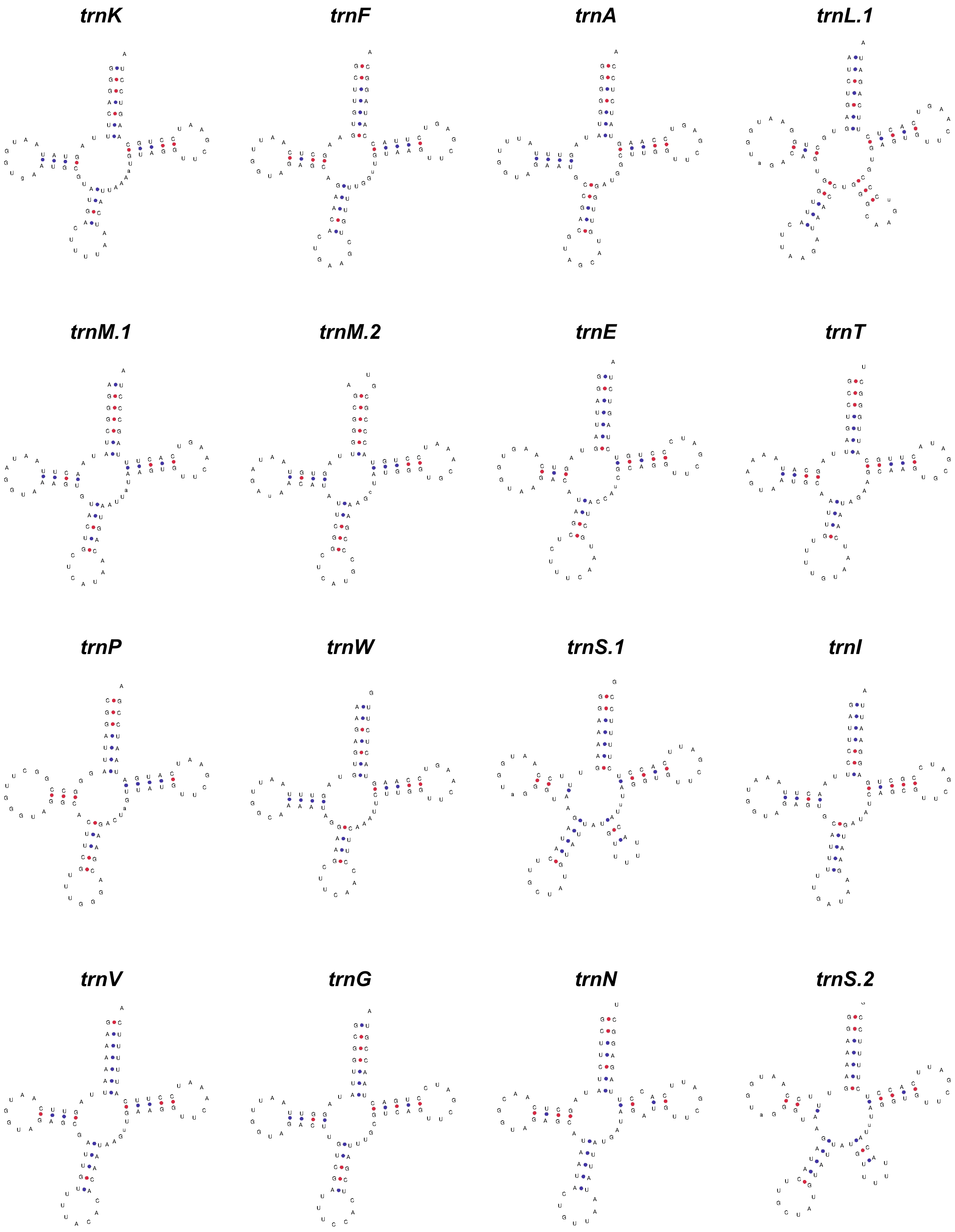
trnK
trnF
trnA
trnL.1
trnM.1
trnM.2
trnE
trnT
trnP
trnW
trnS.1
trnI
trnV
trnG
trnN
trnS.2

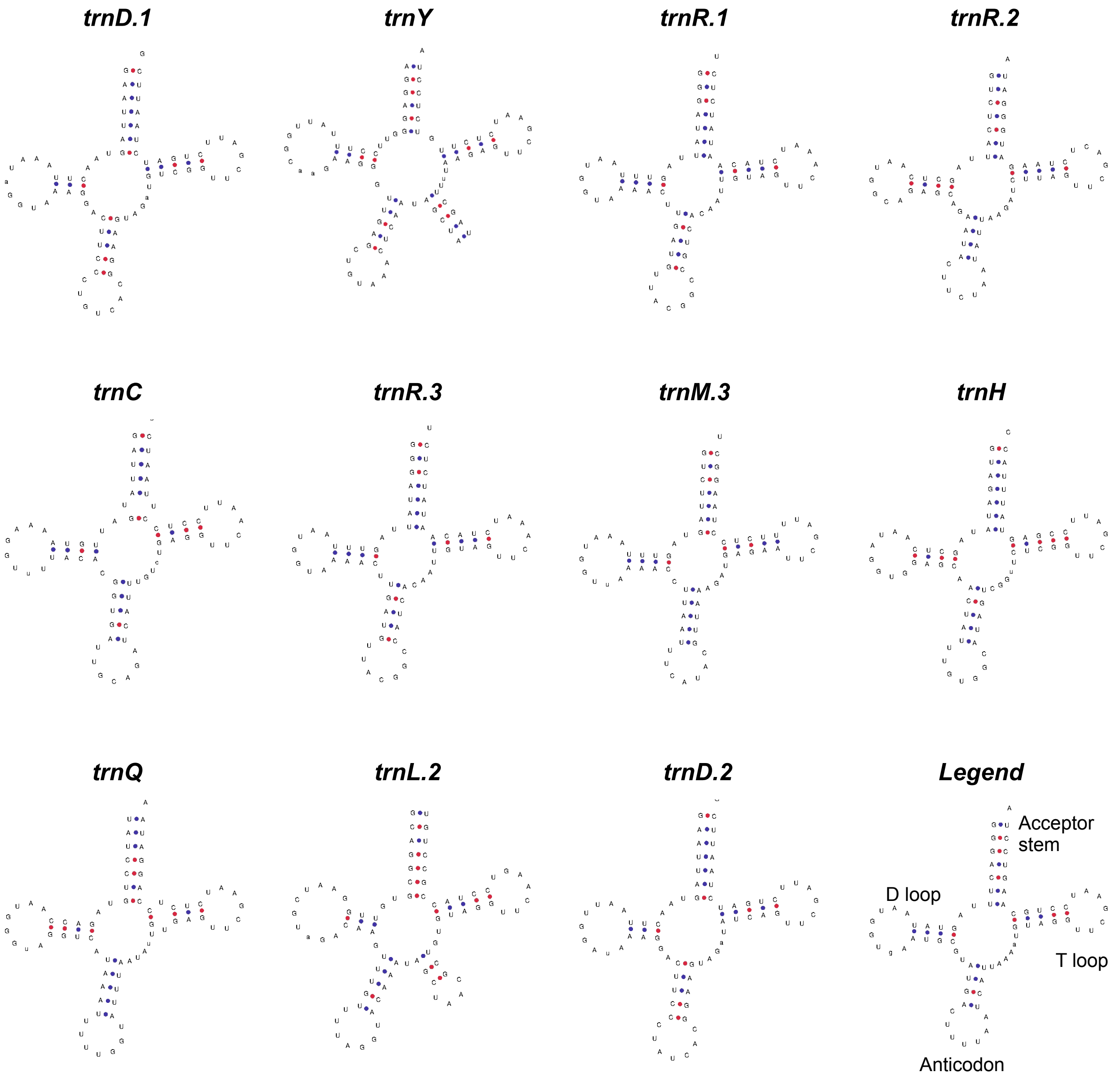
trnD.1
trnY
trnR.1
trnR.2
trnC
trnR.3
trnM.3
trnH
trnQ
trnL.2
trnD.2
Legend
Acceptor stem
D loop
T loop
Anticodon
