# Supplementary material for: Characterization and phylogenetic analysis of the complete mitochondrial genome of the pathogenic fungus Ilyonectria destructans
Source: Sci Rep. 2022 Feb 11;12:2359. doi: 10.1038/s41598-022-05428-z (PMC8837645; doi:10.1038/s41598-022-05428-z)
Supplement: Supplementary file 2 — Supplementary Figure S2. [file 41598_2022_5428_MOESM2_ESM.pdf]

Characterization and phylogenetic analysis of the complete mitochondrial genome of the pathogenic fungus *Ilyonectria destructans*

Piotr Androsiuk*[1], Adam Okorski[2], Łukasz Paukszto[1], Jan Paweł Jastrzębski[1], Sławomir Ciesielski[3], Agnieszka Pszczółkowska[2]

1. Department of Plant Physiology, Genetics and Biotechnology, Faculty of Biology and Biotechnology, University of Warmia and Mazury in Olsztyn, ul. M. Oczapowskiego 1A, 10-719 Olsztyn, Poland.
2. Department of Entomology, Phytopathology and Molecular Diagnostics, Faculty of Agriculture and Forestry, University of Warmia and Mazury in Olsztyn, ul. Prawocheńskiego 17, 10-720 Olsztyn, Poland.
3. University of Warmia and Mazury in Olsztyn, Faculty of Geoengineering, Department of Environmental Biotechnology, Słoneczna 45G, 10-719 Olsztyn, Poland.

* corresponding author – piotr.androsiuk@uwm.edu.pl

Figure S2. Sequence identity plots (mVISTA) among 15 Nectriaceae representatives, using *I. destructans* as reference genome. Arrows indicate the annotated genes and their transcriptional direction. Genome regions are color coded as coding and non-coding regions (blue and red, respectively). The vertical scale represents the degree of identity, which ranged from 50% to 100%.

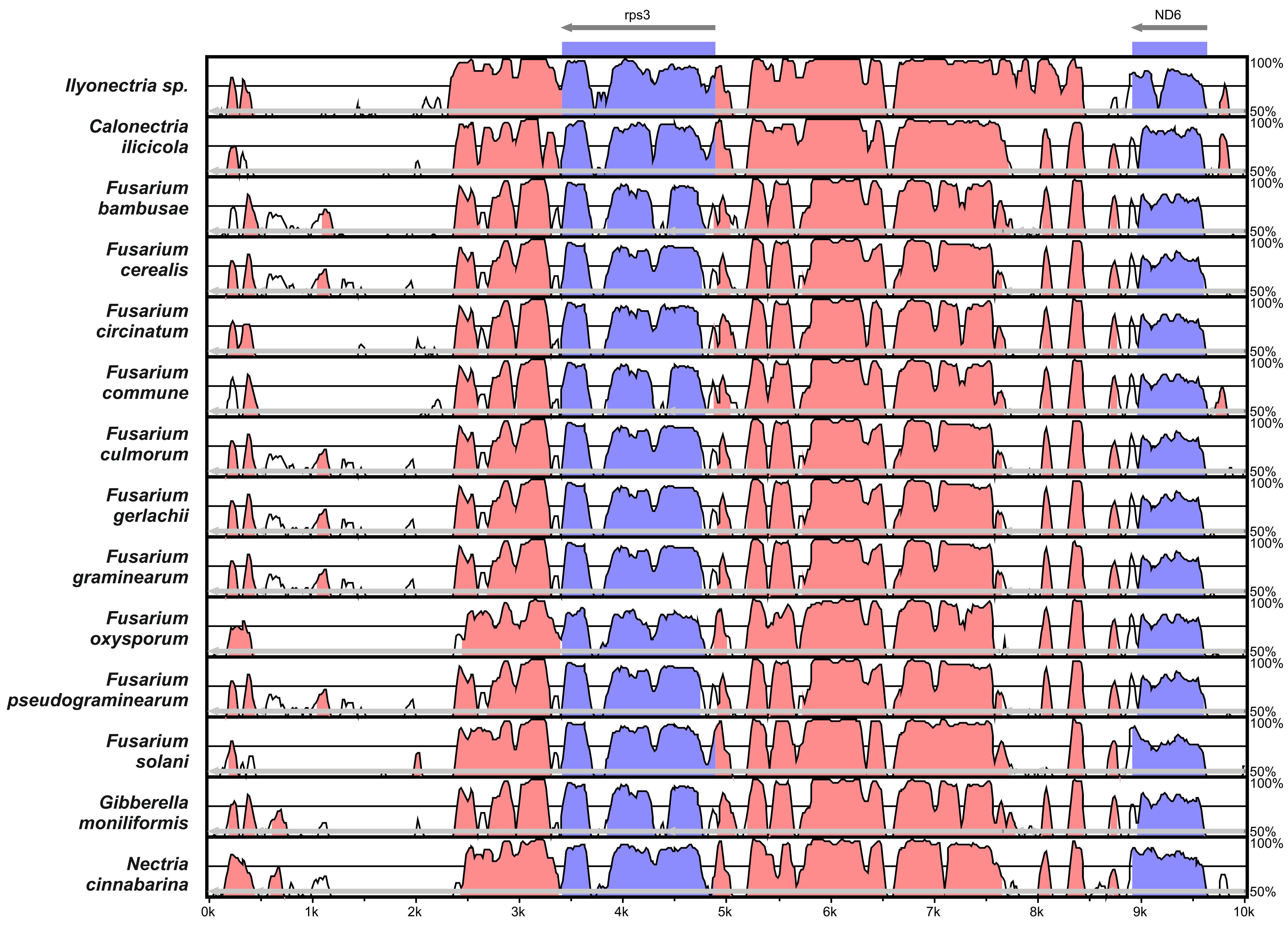

rps3
ND6
Ilyonectria sp.
Calonectria ilicicola
Fusarium bambusae
Fusarium cerealis
Fusarium circinatum
Fusarium commune
Fusarium culmorum
Fusarium gerlachii
Fusarium graminearum
Fusarium oxysporum
Fusarium pseudograminearum
Fusarium solani
Gibberella moniliformis
Nectria cinnabarina
100%
50%
0k
1k
2k
3k
4k
5k
6k
7k
8k
9k
10k

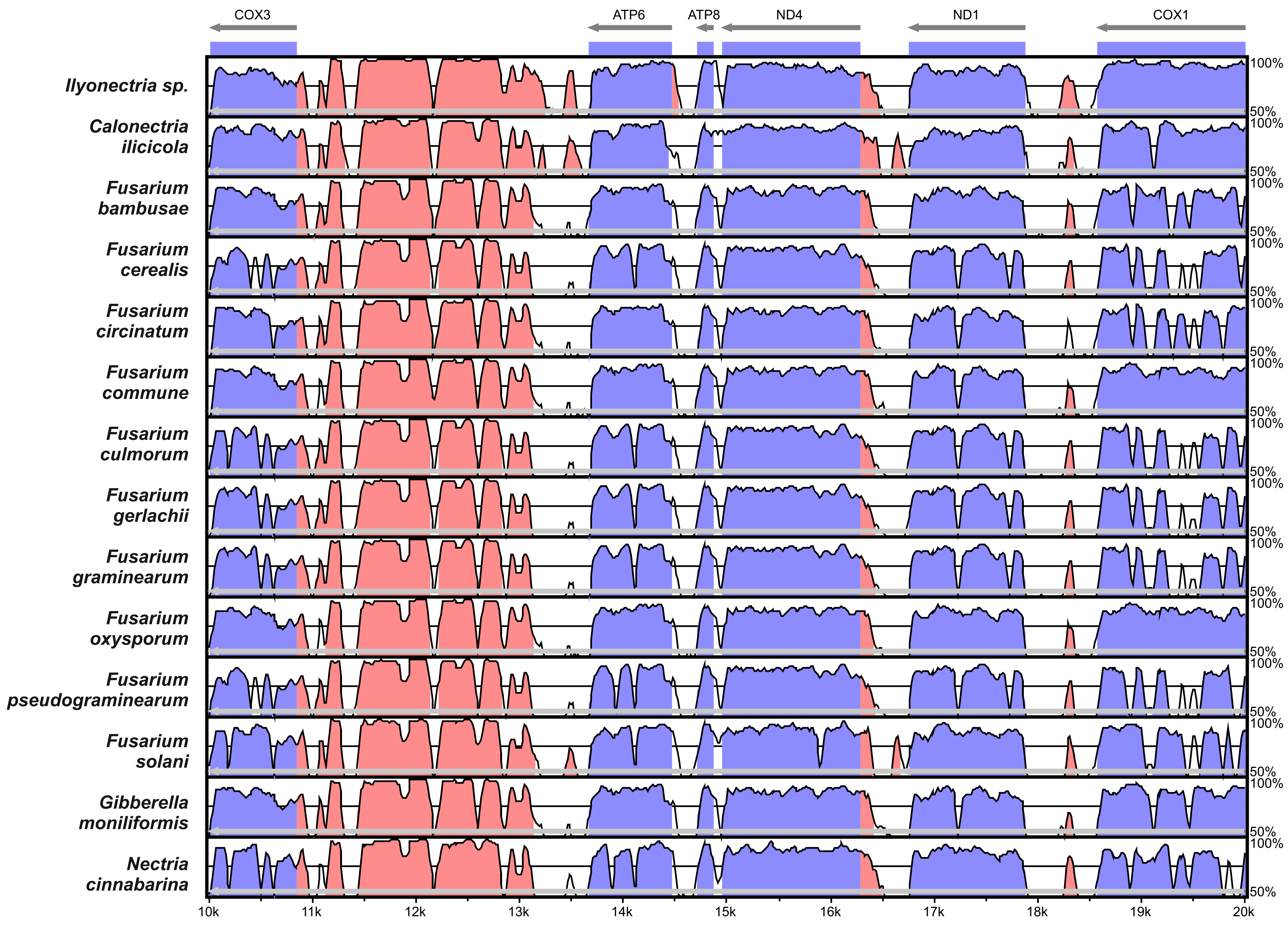

COX3
ATP6
ATP8
ND4
ND1
COX1
Ilyonectria sp.
Calonectria ilicicola
Fusarium bambusae
Fusarium cerealis
Fusarium circinatum
Fusarium commune
Fusarium culmorum
Fusarium gerlachii
Fusarium graminearum
Fusarium oxysporum
Fusarium pseudograminearum
Fusarium solani
Gibberella moniliformis
Nectria cinnabarina
100%
50%
10k
11k
12k
13k
14k
15k
16k
17k
18k
19k
20k

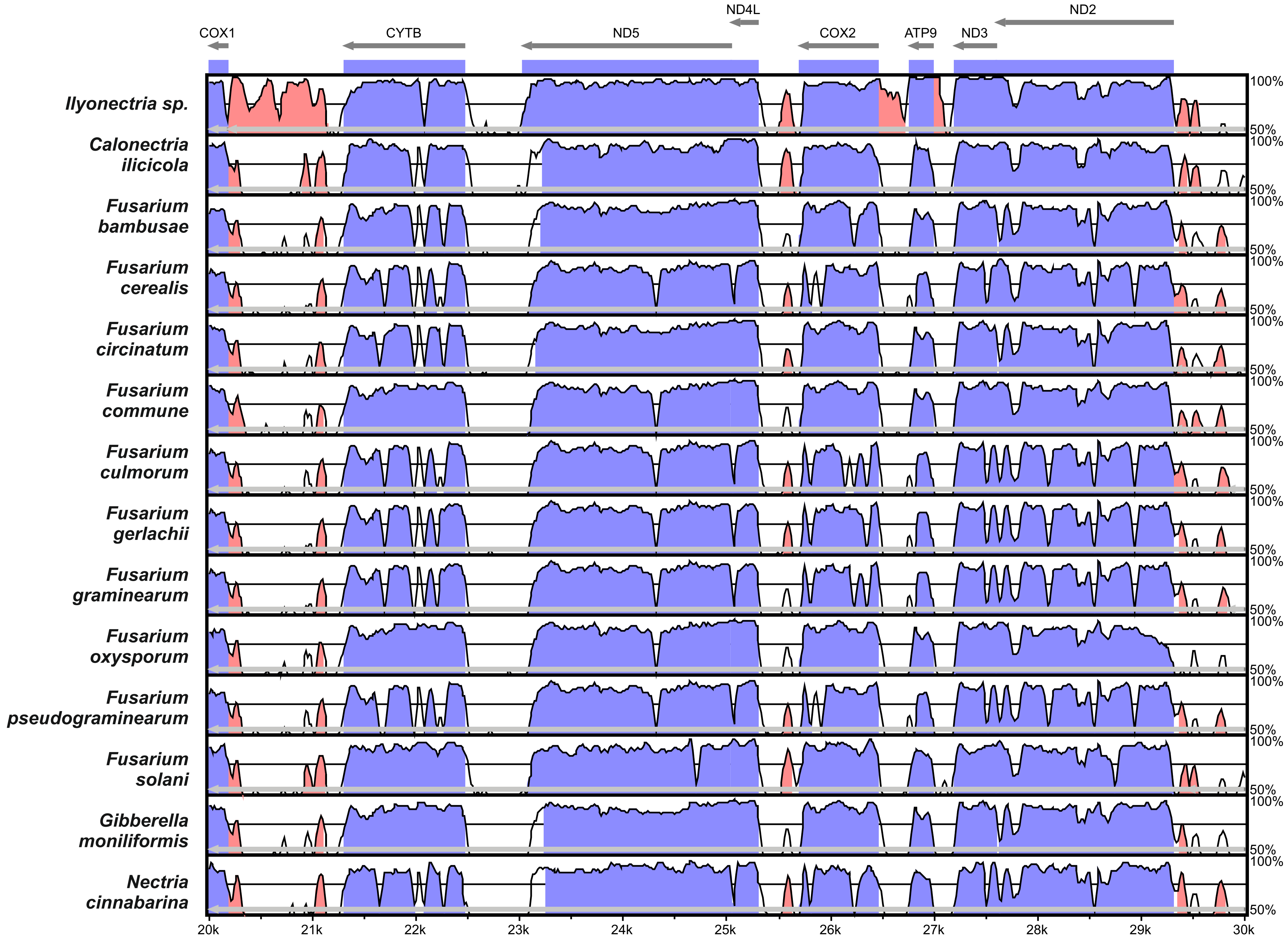

COX1
CYTB
ND5
ND4L
COX2
ATP9
ND3
ND2
Ilyonectria sp.
Calonectria ilicicola
Fusarium bambusae
Fusarium cerealis
Fusarium circinatum
Fusarium commune
Fusarium culmorum
Fusarium gerlachii
Fusarium graminearum
Fusarium oxysporum
Fusarium pseudograminearum
Fusarium solani
Gibberella moniliformis
Nectria cinnabarina
100%
50%
20k
21k
22k
23k
24k
25k
26k
27k
28k
29k
30k

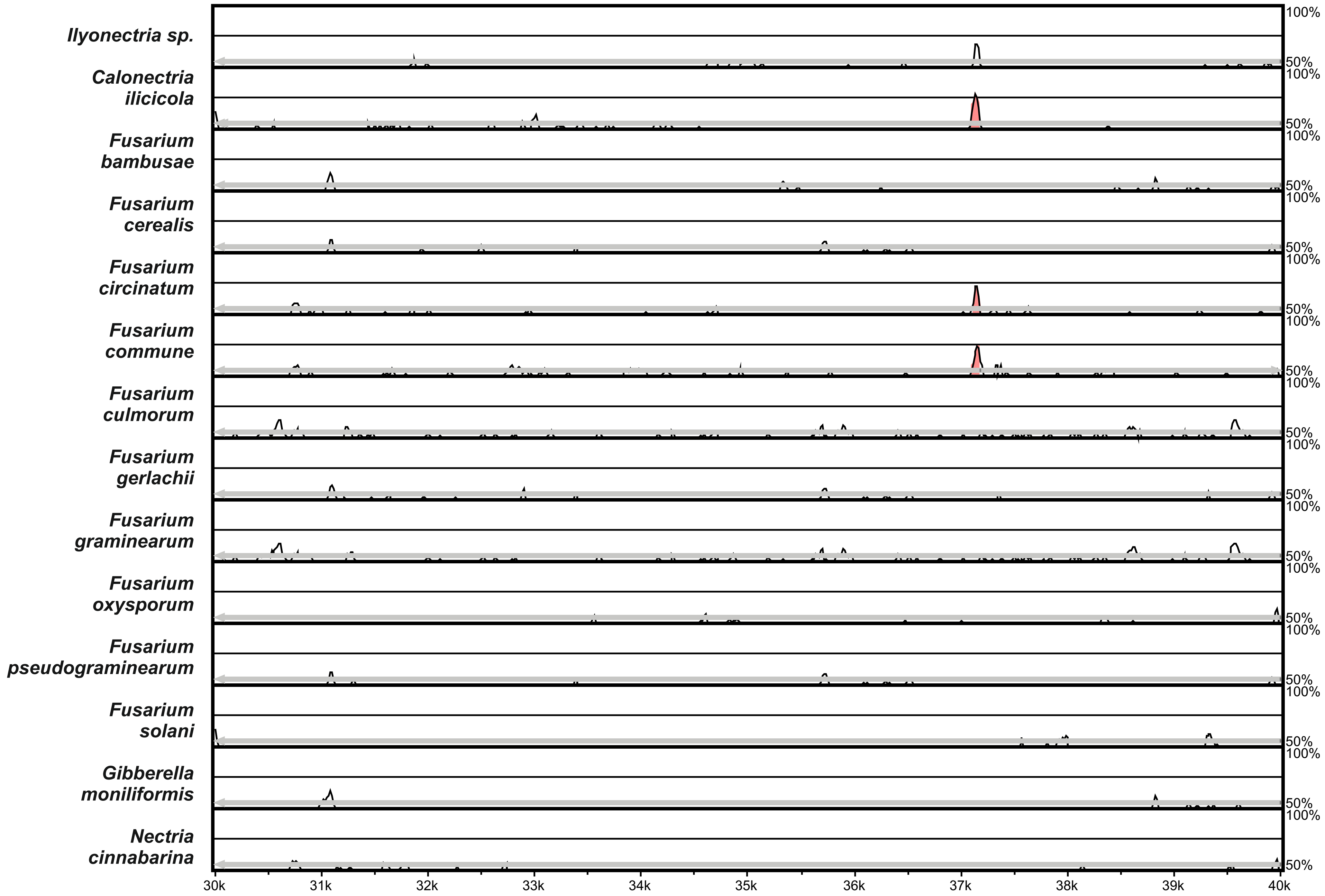

Ilyonectria sp.
Calonectria ilicicola
Fusarium bambusae
Fusarium cerealis
Fusarium circinatum
Fusarium commune
Fusarium culmorum
Fusarium gerlachii
Fusarium graminearum
Fusarium oxysporum
Fusarium pseudograminearum
Fusarium solani
Gibberella moniliformis
Nectria cinnabarina
100%
50%
30k
31k
32k
33k
34k
35k
36k
37k
38k
39k
40k

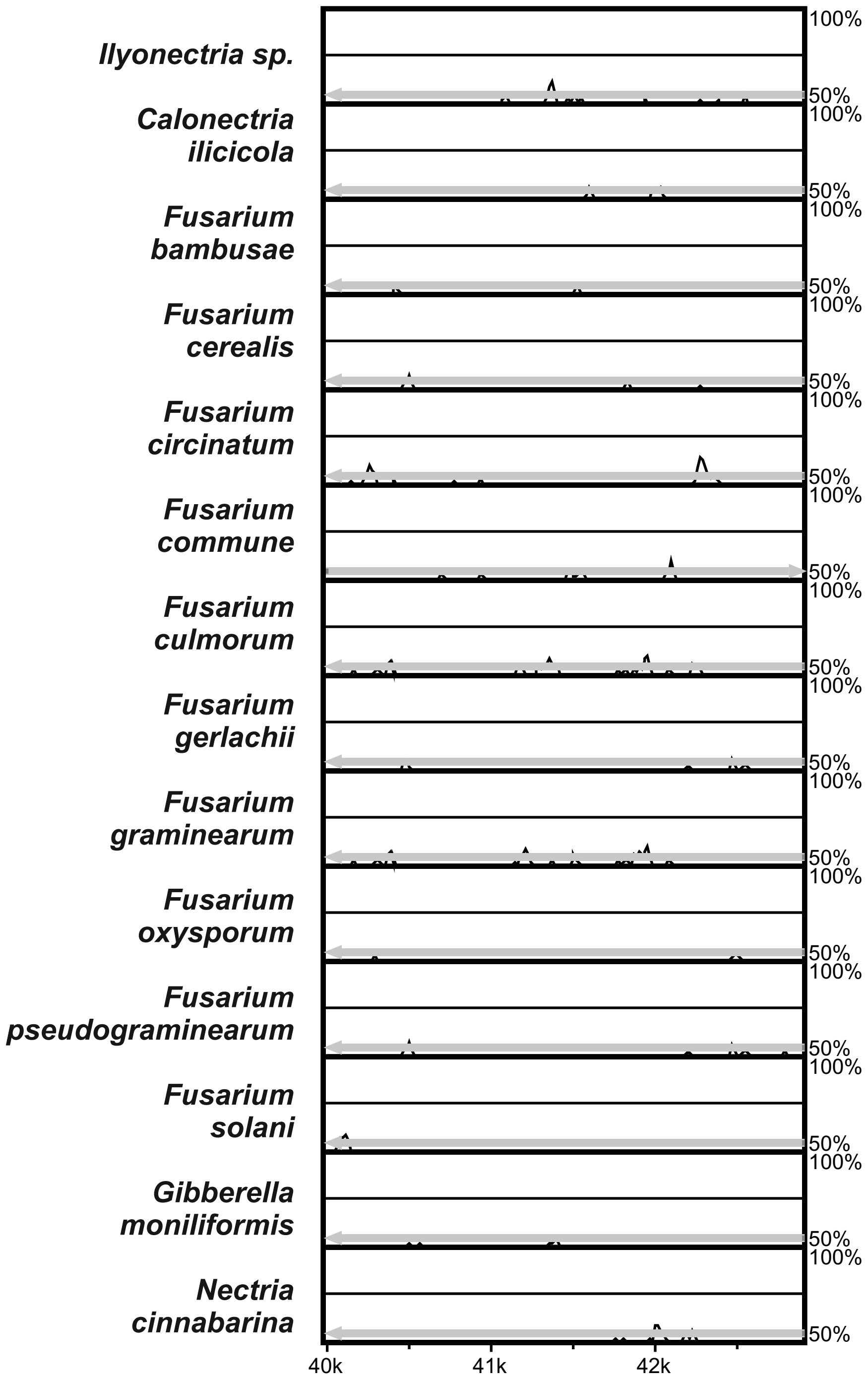

Ilyonectria sp.
Calonectria ilicicola
Fusarium bambusae
Fusarium cerealis
Fusarium circinatum
Fusarium commune
Fusarium culmorum
Fusarium gerlachii
Fusarium graminearum
Fusarium oxysporum
Fusarium pseudograminearum
Fusarium solani
Gibberella moniliformis
Nectria cinnabarina
100%
50%
40k
41k
42k
