# Supplementary material for: Characterization and phylogenetic analysis of the complete mitochondrial genome of the pathogenic fungus Ilyonectria destructans
Source: Sci Rep. 2022 Feb 11;12:2359. doi: 10.1038/s41598-022-05428-z (PMC8837645; doi:10.1038/s41598-022-05428-z)
Supplement: Supplementary file 3 — Supplementary Figure S3. [file 41598_2022_5428_MOESM3_ESM.pdf]

Characterization and phylogenetic analysis of the complete mitochondrial genome of the pathogenic fungus *Ilyonectria destructans*

Piotr Androsiuk*[1], Adam Okorski[2], Łukasz Paukszto[1], Jan Paweł Jastrzębski[1], Sławomir Ciesielski[3], Agnieszka Pszczółkowska[2]

1. Department of Plant Physiology, Genetics and Biotechnology, Faculty of Biology and Biotechnology, University of Warmia and Mazury in Olsztyn, ul. M. Oczapowskiego 1A, 10-719 Olsztyn, Poland.
2. Department of Entomology, Phytopathology and Molecular Diagnostics, Faculty of Agriculture and Forestry, University of Warmia and Mazury in Olsztyn, ul. Prawocheńskiego 17, 10-720 Olsztyn, Poland.
3. University of Warmia and Mazury in Olsztyn, Faculty of Geoengineering, Department of Environmental Biotechnology, Słoneczna 45G, 10-719 Olsztyn, Poland.

* corresponding author – piotr.androsiuk@uwm.edu.pl

Figure S3. A MAUVE alignment of *Calonectria ilicicola* (NC_046826), *Fusarium bambusae* (NC_044490), *F. cerealis* (NC_046567), *F. circinatum* (NC_022681), *F. commune* (NC_036106), *F. culmorum* (NC_026993), *F. gerlachii* (NC_025928), *F. greminearum* (NC_009493), *F. pseudograminearum* (NC_046566), *F. solani* (NC_016680), *F. oxysporium* (NC_017930), *Giberella moniliformis* (NC_016687), *Ilyonectria destructans* (NC_030340), *Ilyonectria sp.* (MH924828) and *Nectria cinnabarina* (NC_030252) mitochondrial genomes showing high synteny of the mitochondrial genomes of the fifteen species. The *I. destructans* mitogenome is shown at top as the reference. Within each of the alignment, local collinear blocks are represented by blocks of the same color connected by lines.

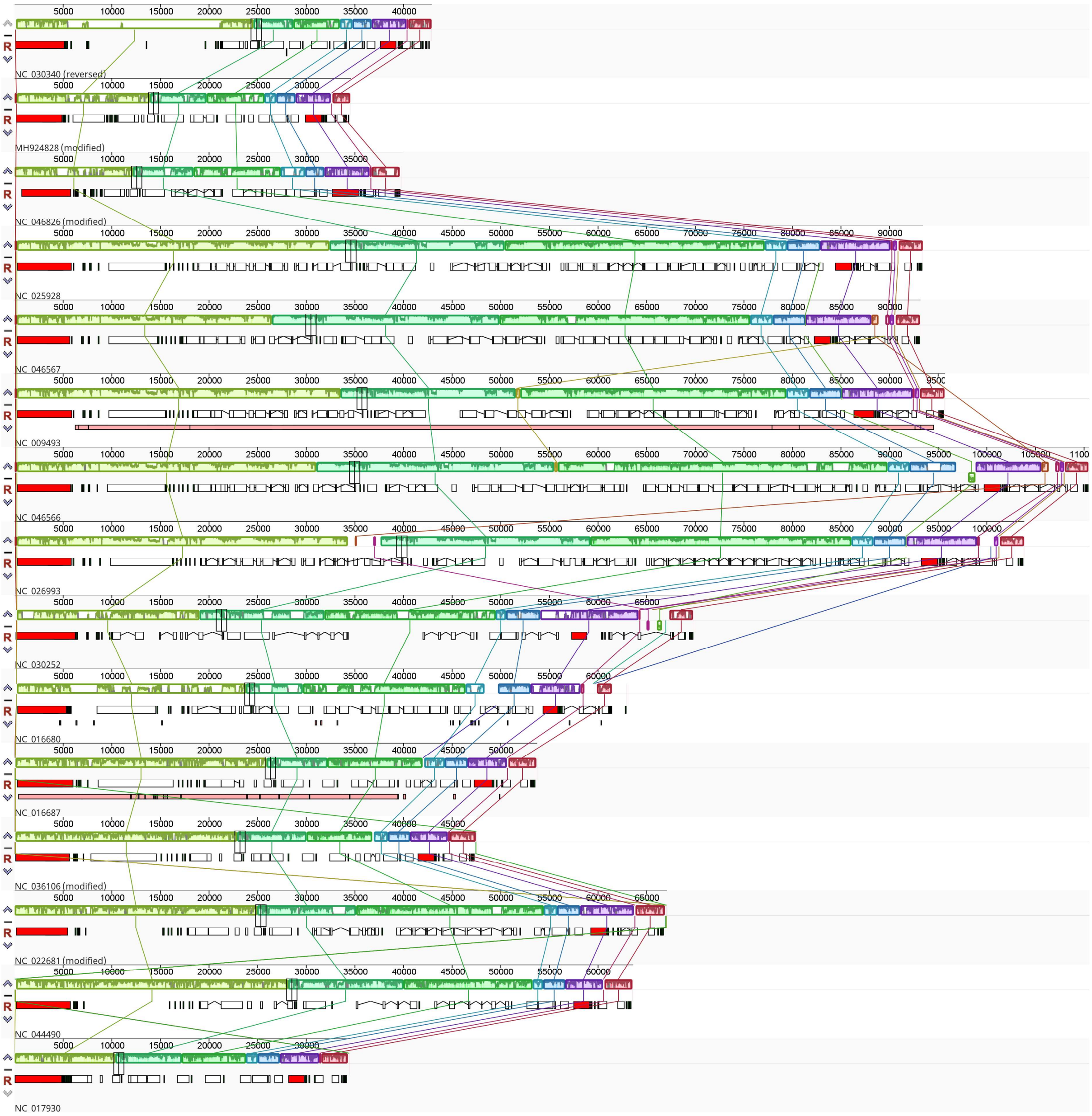
NC_030340 (reversed)
MH924828 (modified)
NC_046826 (modified)
NC_025928
NC_046567
NC_009493
NC_046566
NC_026993
NC_030252
NC_016680
NC_016687
NC_036106 (modified)
NC_022681 (modified)
NC_044490
NC_017930
