# Supplementary material for: Characterization and phylogenetic analysis of the complete mitochondrial genome of the pathogenic fungus Ilyonectria destructans
Source: Sci Rep. 2022 Feb 11;12:2359. doi: 10.1038/s41598-022-05428-z (PMC8837645; doi:10.1038/s41598-022-05428-z)
Supplement: Supplementary file 4 — Supplementary Table S1. [file 41598_2022_5428_MOESM4_ESM.docx]

**Characterization and phylogenetic analysis of the complete mitochondrial genome of the pathogenic fungus *Ilyonectria destructans***

Piotr Androsiuk*^1^, Adam Okorski^2^, Łukasz Paukszto^1^, Jan Paweł Jastrzębski^1^, Sławomir Ciesielski^3^, Agnieszka Pszczółkowska^2^

1. Department of Plant Physiology, Genetics and Biotechnology, Faculty of Biology and Biotechnology, University of Warmia and Mazury in Olsztyn, ul. M. Oczapowskiego 1A, 10-719 Olsztyn, Poland.

2. Department of Entomology, Phytopathology and Molecular Diagnostics, Faculty of Agriculture and Forestry, University of Warmia and Mazury in Olsztyn, ul. Prawocheńskiego 17, 10-720 Olsztyn, Poland.

3. Department of Environmental Biotechnology, Faculty of Geoengineering, University of Warmia and Mazury in Olsztyn, Słoneczna 45G, 10-719 Olsztyn, Poland.

* corresponding author – piotr.androsiuk@uwm.edu.pl

**Table S1.** Codon usage in *Ilyonectria destructans* mitochondrial genome.

| **Codon** | **Amino Acid** | **%** | **Count** | **Freq** | **Codon** | **Amino Acid** | **%** | **Count** | **Freq** |
| --- | --- | --- | --- | --- | --- | --- | --- | --- | --- |
| **GCA** | Ala | 32.0% | 134 | 0.0151 | **AAT** | Asn | 81.9% | 546 | 0.0614 |
| **GCC** |  | 10.7% | 45 | 0.0051 | **CCA** | Pro | 23.6% | 66 | 0.0074 |
| **GCG** |  | 5.0% | 21 | 0.0024 | **CCC** |  | 9.6% | 27 | 0.0030 |
| **GCT** |  | 52.3% | 219 | 0.0246 | **CCG** |  | 5.4% | 15 | 0.0017 |
| **TGC** | Cys | 24.3% | 17 | 0.0019 | **CCT** |  | 61.4% | 172 | 0.0193 |
| **TGT** |  | 75.7% | 53 | 0.0060 | **CAA** | Gln | 84.2% | 155 | 0.0174 |
| **GAC** | Asp | 13.7% | 43 | 0.0048 | **CAG** |  | 15.8% | 29 | 0.0033 |
| **GAT** |  | 86.3% | 270 | 0.0303 | **AGA** | Arg | 69.7% | 191 | 0.0215 |
| **GAA** | Glu | 88.0% | 309 | 0.0347 | **AGG** |  | 8.0% | 22 | 0.0025 |
| **GAG** |  | 12.0% | 42 | 0.0047 | **CGA** |  | 4.4% | 12 | 0.0013 |
| **TTC** | Phe | 28.1% | 174 | 0.0196 | **CGC** |  | 1.1% | 3 | 0.0003 |
| **TTT** |  | 71.9% | 445 | 0.0500 | **CGG** |  | 1.1% | 3 | 0.0003 |
| **GGA** | Gly | 35.6% | 170 | 0.0191 | **CGT** |  | 15.7% | 43 | 0.0048 |
| **GGC** |  | 3.8% | 18 | 0.0020 | **AGC** | Ser | 10.9% | 80 | 0.0090 |
| **GGG** |  | 9.4% | 45 | 0.0051 | **AGT** |  | 32.1% | 236 | 0.0265 |
| **GGT** |  | 51.2% | 244 | 0.0274 | **TCA** |  | 20.9% | 154 | 0.0173 |
| **CAC** | His | 16.5% | 27 | 0.0030 | **TCC** |  | 2.9% | 21 | 0.0024 |
| **CAT** |  | 83.5% | 137 | 0.0154 | **TCG** |  | 2.4% | 18 | 0.0020 |
| **ATA** | Ile | 59.8% | 543 | 0.0610 | **TCT** |  | 30.8% | 227 | 0.0255 |
| **ATC** |  | 6.6% | 60 | 0.0067 | **ACA** | Thr | 38.3% | 172 | 0.0193 |
| **ATT** |  | 33.6% | 305 | 0.0343 | **ACC** |  | 7.3% | 33 | 0.0037 |
| **AAA** | Lys | 84.0% | 437 | 0.0491 | **ACG** |  | 6.2% | 28 | 0.0031 |
| **AAG** |  | 16.0% | 83 | 0.0093 | **ACT** |  | 48.1% | 216 | 0.0243 |
| **CTA** | Leu | 9.6% | 104 | 0.0117 | **GTA** | Val | 52.6% | 269 | 0.0302 |
| **CTC** |  | 0.7% | 8 | 0.0009 | **GTC** |  | 3.1% | 16 | 0.0018 |
| **CTG** |  | 1.7% | 19 | 0.0021 | **GTG** |  | 11.0% | 56 | 0.0063 |
| **CTT** |  | 8.5% | 93 | 0.0105 | **GTT** |  | 33.3% | 170 | 0.0191 |
| **TTA** |  | 72.2% | 786 | 0.0883 | **TGA** | TRP | 91.3% | 95 | 0.0107 |
| **TTG** |  | 7.2% | 78 | 0.0088 | **TGG** |  | 8.7% | 9 | 0.0010 |
| **ATA** | Met | 0.4% | 1 | 0.0001 | **TAC** | Tyr | 18.9% | 97 | 0.0109 |
| **ATG** |  | 97.8% | 219 | 0.0246 | **TAT** |  | 81.1% | 415 | 0.0466 |
| **ATT** |  | 0.4% | 1 | 0.0001 | **TAA** | * | 75.0% | 21 | 0.0024 |
| **TTA** |  | 1.3% | 3 | 0.0003 | **TAG** |  | 25.0% | 7 | 0.0008 |
| **AAC** | Asn | 18.1% | 121 | 0.0136 |  |  |  |  |  |
