# Supplementary material for: Characterization and phylogenetic analysis of the complete mitochondrial genome of the pathogenic fungus Ilyonectria destructans
Source: Sci Rep. 2022 Feb 11;12:2359. doi: 10.1038/s41598-022-05428-z (PMC8837645; doi:10.1038/s41598-022-05428-z)
Supplement: Supplementary file 5 — Supplementary Table S2. [file 41598_2022_5428_MOESM5_ESM.docx]

**Characterization and phylogenetic analysis of the complete mitochondrial genome of the pathogenic fungus *Ilyonectria destructans***

Piotr Androsiuk*^1^, Adam Okorski^2^, Łukasz Paukszto^1^, Jan Paweł Jastrzębski^1^, Sławomir Ciesielski^3^, Agnieszka Pszczółkowska^2^

1. Department of Plant Physiology, Genetics and Biotechnology, Faculty of Biology and Biotechnology, University of Warmia and Mazury in Olsztyn, ul. M. Oczapowskiego 1A, 10-719 Olsztyn, Poland.

2. Department of Entomology, Phytopathology and Molecular Diagnostics, Faculty of Agriculture and Forestry, University of Warmia and Mazury in Olsztyn, ul. Prawocheńskiego 17, 10-720 Olsztyn, Poland.

3. Department of Environmental Biotechnology, Faculty of Geoengineering, University of Warmia and Mazury in Olsztyn, Słoneczna 45G, 10-719 Olsztyn, Poland.

* corresponding author – piotr.androsiuk@uwm.edu.pl

**Table S2a**. List of repeated sequences in the mitochondrial genomes of *Ilyonectria destructans*.

| **Species** | **Repeat length (bp)** | **Strat site of repeat A** | **Repeat A location** | **Strat site of repeat B** | **Repeat B location** | **Repeat type** |
| --- | --- | --- | --- | --- | --- | --- |
| *Ilyonectria destructans* | 98 | 16,596 | trnR | 25,543 | trnR | F |
|  | 57 | 16,637 | trnR | 25,584 | trnR | F |
|  | 54 | 11,130 | trnD | 37,109 | trnD | F |
|  | 45 | 13,263 | IGS (rns-atp6) | 25,360 | IGS (nad4L-trnR) | P |
|  | 35 | 11,170 | trnD | 37,149 | trnD | F |
|  | 35 | 9926 | IGS (trnG-cox3) | 9926 | IGS (trnG-cox3) | R |
|  | 34 | 9961 | IGS (trnG-cox3) | 25,367 | IGS (nad4L-trnR) | P |
|  | 34 | 9961 | IGS (trnG-cox3) | 13,267 | IGS (rns-atp6) | F |
|  | 33 | 18,142 | IGS (nad1-trnR) | 25,364 | IGS (nad4L-trnR) | P |
|  | 33 | 13,271 | IGS (rns-atp6) | 18,142 | IGS (nad1-trnR) | F |
|  | 32 | 11,152 | trnD | 37,131 | trnD | F |
|  | 32 | 7780 | IGS (rnl-trnP) | 13,270 | IGS (rns-atp6) | F |
|  | 32 | 7780 | IGS (rnl-trnP) | 25,366 | IGS (nad4L-trnR) | P |
|  | 32 | 2115 | IGS (trnL-trnM) | 29,870 | IGS (trnQ-orf326) | F |
|  | 31 | 8188 | IGS (trnP-trnW) | 25,372 | IGS (nad4L-trnR) | P |
|  | 31 | 8188 | IGS (trnP-trnW) | 13,265 | IGS (rns-atp6) | F |
|  | 31 | 7818 | IGS (rnl-trnP) | 20,387 | IGS (cox1-trnC) | P |
|  | 31 | 7781 | IGS (rnl-trnP) | 18,142 | IGS (nad1-trnR) | F |
|  | 31 | 7780 | IGS (rnl-trnP) | 9964 | IGS (trnG-cox3) | F |
|  | 31 | 6327 | rnl | 6327 | rnl | R |
|  | 30 | 16,510 | IGS (nad4-trnR) | 37,214 | IGS (trnD-orf179) | F |
|  | 30 | 9965 | IGS (trnG-cox3) | 18,142 | IGS (nad1-trnR) | F |
|  | 30 | 8191 | IGS (trnP-trnW) | 21,148 | IGS (trnC-cob) | P |
|  | 30 | 2128 | IGS (trnL-trnM) | 7781 | IGS (rnl-trnP) | F |
|  | 30 | 2128 | IGS (trnL-trnM) | 13,271 | IGS (rns-atp6) | F |
|  | 30 | 2128 | IGS (trnL-trnM) | 18,142 | IGS (nad1-trnR) | F |
|  | 30 | 2128 | IGS (trnL-trnM) | 25,367 | IGS (nad4L-trnR) | P |
|  | 30 | 2128 | IGS (trnL-trnM) | 9965 | IGS (trnG-cox3) | F |

IGS (rns-atp6) means spacer between *rns* and *atp6*, P means palindromic match, F means forward (direct) match, and R means reverse match.
