# Supplementary material for: Characterization and phylogenetic analysis of the complete mitochondrial genome of the pathogenic fungus Ilyonectria destructans
Source: Sci Rep. 2022 Feb 11;12:2359. doi: 10.1038/s41598-022-05428-z (PMC8837645; doi:10.1038/s41598-022-05428-z)
Supplement: Supplementary file 6 — Supplementary Table S2. [file 41598_2022_5428_MOESM6_ESM.docx]

**Characterization and phylogenetic analysis of the complete mitochondrial genome of the pathogenic fungus *Ilyonectria destructans***

Piotr Androsiuk*^1^, Adam Okorski^2^, Łukasz Paukszto^1^, Jan Paweł Jastrzębski^1^, Sławomir Ciesielski^3^, Agnieszka Pszczółkowska^2^

1. Department of Plant Physiology, Genetics and Biotechnology, Faculty of Biology and Biotechnology, University of Warmia and Mazury in Olsztyn, ul. M. Oczapowskiego 1A, 10-719 Olsztyn, Poland.

2. Department of Entomology, Phytopathology and Molecular Diagnostics, Faculty of Agriculture and Forestry, University of Warmia and Mazury in Olsztyn, ul. Prawocheńskiego 17, 10-720 Olsztyn, Poland.

3. Department of Environmental Biotechnology, Faculty of Geoengineering, University of Warmia and Mazury in Olsztyn, Słoneczna 45G, 10-719 Olsztyn, Poland.

* corresponding author – piotr.androsiuk@uwm.edu.pl

**Table S2b**. List of repeated sequences in the mitochondrial genomes of *Ilyonectria sp*.

| **Species** | **Repeat length (bp)** | **Strat site of repeat A** | **Repeat A location** | **Strat site of repeat B** | **Repeat B location** | **Repeat type** |
| --- | --- | --- | --- | --- | --- | --- |
| *Ilyonectria sp.* | 34 | 18,197 | IGS (trnM-CAT-trnL-TAA) | 18,243 | IGS (trnM-CAT-trnL-TAA) | F |
|  | 32 | 27,488 | IGS (cox2-trnR-ACG) | 27,592 | trnR-ACG | F |
|  | 32 | 12,716 | IGS (trnP-TGG-rnl) | 12,888 | IGS (trnP-TGG-rnl) | F |
|  | 32 | 12,709 | IGS (trnP-TGG-rnl) | 22,439 | IGS (trnF-GAA-trnK-TTT) | F |
|  | 32 | 12,448 | IGS (trnW-TCA-trnP-TGG) | 22,450 | IGS (trnF-GAA-trnK-TTT) | F |
|  | 32 | 2146 | IGS (trnR-TCT-ORF174) | 2192 | IGS (trnR-TCT-ORF174) | F |
|  | 32 | 1752 | IGS (cox1-trnR-TCT) | 1752 | IGS (cox1-trnR-TCT) | P |
|  | 31 | 34,498 | IGS (trnC-GCA-cox1) | 34,553 | IGS (trnC-GCA-cox1) | F |
|  | 31 | 12,693 | IGS (trnP-TGG-rnl) | 34,019 | IGS (trnC-GCA-cox1) | F |
|  | 30 | 27,323 | IGS (cox2-trnR-ACG) | 27,329 | IGS (cox2-trnR-ACG) | F |
|  | 30 | 25,697 | IGS (nad3-atp9) | 26,211 | IGS (atp9-cox2) | F |
|  | 30 | 6529 | IGS (atp8-atp6) | 6529 | IGS (atp8-atp6) | P |
|  | 30 | 0 | cox1 | 34,529 | IGS (trnC-GCA-cox1) | F |

IGS (*atp8-atp6*) means spacer between *atp8* and *atp6*, P means palindromic match, F means forward (direct) match, and R means reverse match.
