# Supplementary material for: Characterization and phylogenetic analysis of the complete mitochondrial genome of the pathogenic fungus Ilyonectria destructans
Source: Sci Rep. 2022 Feb 11;12:2359. doi: 10.1038/s41598-022-05428-z (PMC8837645; doi:10.1038/s41598-022-05428-z)
Supplement: Supplementary file 7 — Supplementary Table S3. [file 41598_2022_5428_MOESM7_ESM.docx]

**Characterization and phylogenetic analysis of the complete mitochondrial genome of the pathogenic fungus *Ilyonectria destructans***

Piotr Androsiuk*^1^, Adam Okorski^2^, Łukasz Paukszto^1^, Jan Paweł Jastrzębski^1^, Sławomir Ciesielski^3^, Agnieszka Pszczółkowska^2^

1. Department of Plant Physiology, Genetics and Biotechnology, Faculty of Biology and Biotechnology, University of Warmia and Mazury in Olsztyn, ul. M. Oczapowskiego 1A, 10-719 Olsztyn, Poland.

2. Department of Entomology, Phytopathology and Molecular Diagnostics, Faculty of Agriculture and Forestry, University of Warmia and Mazury in Olsztyn, ul. Prawocheńskiego 17, 10-720 Olsztyn, Poland.

3. Department of Environmental Biotechnology, Faculty of Geoengineering, University of Warmia and Mazury in Olsztyn, Słoneczna 45G, 10-719 Olsztyn, Poland.

* corresponding author – piotr.androsiuk@uwm.edu.pl

**Table S3a.** Distribution of SSR in the *Ilyonectria destructans* mt genome

| **Type** | **Repeat unit** | **Length** | **Start** | **End** | **Location** |
| --- | --- | --- | --- | --- | --- |
| Dinucleotide Repeat | AT | 15 | 6336 | 6350 | large subunit ribosomal RNA |
|  | AC | 14 | 8825 | 8838 | IGS (trnI-GAU-trnV-UAC) |
|  | AT | 12 | 35,853 | 35,864 | IGS (orf1210-orf325) |
| Trinucleotide Repeat | ATA | 14 | 30,996 | 31,009 | orf 326 |
|  | ATT | 14 | 1409 | 1422 | orf 340 |
|  | ATA | 13 | 42,226 | 42,238 | orf210 |
|  | AAT | 13 | 37,334 | 37,346 | IGS (trnD-AUC-orf179) |
|  | ATT | 12 | 31,844 | 31,855 | IGS (orf180-orf1210) |
| Tetranucleotide Repeat | TGCG | 15 | 20,470 | 20,484 | IGS (cox1-trnC-GCA) |
|  | TTAA | 12 | 29,334 | 29,345 | IGS (nad2-trnM-CAU) |
|  | AGCA | 12 | 20,498 | 20,509 | IGS (cox1-trnC-GCA) |
| Hexanucleotide Repeat | AAGCTA | 24 | 16,527 | 16,550 | IGS (nad4-trnR-ACG) |
|  | TTATTC | 19 | 2185 | 2203 | IGS (trnL-UAA-trnM-CAU) |
|  | TTTTCT | 18 | 18,137 | 18,154 | IGS (nad1-trnR-UCU) |
