# Supplementary material for: Characterization and phylogenetic analysis of the complete mitochondrial genome of the pathogenic fungus Ilyonectria destructans
Source: Sci Rep. 2022 Feb 11;12:2359. doi: 10.1038/s41598-022-05428-z (PMC8837645; doi:10.1038/s41598-022-05428-z)
Supplement: Supplementary file 8 — Supplementary Table S3. [file 41598_2022_5428_MOESM8_ESM.docx]

**Characterization and phylogenetic analysis of the complete mitochondrial genome of the pathogenic fungus *Ilyonectria destructans***

Piotr Androsiuk*^1^, Adam Okorski^2^, Łukasz Paukszto^1^, Jan Paweł Jastrzębski^1^, Sławomir Ciesielski^3^, Agnieszka Pszczółkowska^2^

1. Department of Plant Physiology, Genetics and Biotechnology, Faculty of Biology and Biotechnology, University of Warmia and Mazury in Olsztyn, ul. M. Oczapowskiego 1A, 10-719 Olsztyn, Poland.

2. Department of Entomology, Phytopathology and Molecular Diagnostics, Faculty of Agriculture and Forestry, University of Warmia and Mazury in Olsztyn, ul. Prawocheńskiego 17, 10-720 Olsztyn, Poland.

3. Department of Environmental Biotechnology, Faculty of Geoengineering, University of Warmia and Mazury in Olsztyn, Słoneczna 45G, 10-719 Olsztyn, Poland.

* corresponding author – piotr.androsiuk@uwm.edu.pl

**Table S3b.** Distribution of SSR in the *Ilyonectria sp.* mt genome

| **Type** | **Repeat unit** | **Length** | **Start** | **End** | **Location** |
| --- | --- | --- | --- | --- | --- |
| Dinucleotide Repeat | AC | 15 | 7591 | 7605 | IGS (atp6-rns) |
|  | AC | 13 | 27,864 | 27,876 | IGS (trnR-ACG-nad4L) |
| Trinucleotide Repeat | AAT | 12 | 328 | 339 | cox1 |
|  | ATC | 12 | 11,069 | 11,080 | cox3 |
| Tetranucleotide Repeat | AAAT | 15 | 3340 | 3354 | IGS (orf174-nad1) |
|  | AAGC | 14 | 6161 | 6174 | IGS (nad4-atp8) |
|  | ATGC | 12 | 7567 | 7578 | IGS (atp6-rns) |
|  | ATGC | 12 | 22,064 | 22,075 | IGS (orf1076-trnA-TGC) |
|  | AAAT | 12 | 30,727 | 30,738 | IGS (nad5-cob) |
| Hexanucleotide Repeat | AAACGT | 23 | 27,324 | 27,346 | IGS (cox2-trnR-ACG) |
|  | AGATAT | 20 | 18,982 | 19,001 | orf1076 |
|  | AAATAT | 19 | 32,838 | 32,856 | cob |
