# Supplementary material for: Characterization and phylogenetic analysis of the complete mitochondrial genome of the pathogenic fungus Ilyonectria destructans
Source: Sci Rep. 2022 Feb 11;12:2359. doi: 10.1038/s41598-022-05428-z (PMC8837645; doi:10.1038/s41598-022-05428-z)
Supplement: Supplementary file 10 — Supplementary Table S5. [file 41598_2022_5428_MOESM10_ESM.docx]

**Characterization and phylogenetic analysis of the complete mitochondrial genome of the pathogenic fungus *Ilyonectria destructans***

Piotr Androsiuk*^1^, Adam Okorski^2^, Łukasz Paukszto^1^, Jan Paweł Jastrzębski^1^, Sławomir Ciesielski^3^, Agnieszka Pszczółkowska^2^

1. Department of Plant Physiology, Genetics and Biotechnology, Faculty of Biology and Biotechnology, University of Warmia and Mazury in Olsztyn, ul. M. Oczapowskiego 1A, 10-719 Olsztyn, Poland.

2. Department of Entomology, Phytopathology and Molecular Diagnostics, Faculty of Agriculture and Forestry, University of Warmia and Mazury in Olsztyn, ul. Prawocheńskiego 17, 10-720 Olsztyn, Poland.

3. Department of Environmental Biotechnology, Faculty of Geoengineering, University of Warmia and Mazury in Olsztyn, Słoneczna 45G, 10-719 Olsztyn, Poland.

* corresponding author – piotr.androsiuk@uwm.edu.pl

**Table S5.** List of mitochondrial genomes used in the phylogenetic analysis. Species list arranged alphabetically.

| **Species name** | **Accesion number** |
| --- | --- |
| *Acremonium fuci* | NC_029851 |
| *Beauveria bassiana* | NC_010652 |
| *Beauveria caledonica* | NC_030636 |
| *Beauveria malawiensis* | NC_030635 |
| *Beauveria pseudobassiana* | NC_022708 |
| *Calonectria ilicicola* | NC_046826 |
| *Clonostachys rosea* | NC_036667 |
| *Cordyceps brongniartii* | NC_011194 |
| *Cordyceps cicadae* | NC_041489 |
| *Cordyceps confragosa* | NC_046840 |
| *Cordyceps militaris* | NC_022834 |
| *Epichloe festucae* | NC_032064 |
| *Epichloe typhina* | NC_032063 |
| *Fusarium bambusae* | NC_044490 |
| *Fusarium cerealis* | NC_046567 |
| *Fusarium circinatum* | NC_022681 |
| *Fusarium commune* | NC_036106 |
| *Fusarium culmorum* | NC_026993 |
| *Fusarium gerlachii* | NC_025928 |
| *Fusarium graminearum* | NC_009493 |
| *Fusarium oxysporum* | NC_017930 |
| *Fusarium pseudograminearum* | NC_046566 |
| *Fusarium solani* | NC_016680 |
| *Gibberella moniliformis* | NC_016687 |
| *Hirsutella minnesotensis* | NC_027660 |
| *Hirsutella rhossiliensis* | NC_030164 |
| *Hirsutella thompsonii* | NC_040165 |
| *Hirsutella vermicola* | NC_036610 |
| *Hypomyces aurantius* | NC_030206 |
| *Ilyonectria destructans* | NC_030340 |
| *Ilyonectria sp.* | MH924828 |
| *Lecanicillium muscarium* | NC_004514 |
| *Lecanicillium saksenae* | NC_028330 |
| *Metacordyceps chlamydosporia* | NC_022835 |
| *Metarhizium anisopliae* | NC_008068 |
| *Metarhizium rileyi* | NC_047289 |
| *Nectria cinnabarina* | NC_030252 |
| *Ophiocordyceps sinensis* | NC_034659 |
| *Parengyodontium album* | NC_032302 |
| *Tolypocladium cylindrosporum* | NC_046839 |
| *Tolypocladium inflatum* | NC_036382 |
| *Tolypocladium ophioglossoides* | NC_031384 |
| *Trichoderma hamatum* | NC_036144 |
| *Trichoderma reesei* | NC_003388 |
